# Period-doubling cascade to chaos and optimal quadratic harvesting in a prey–predator–scavenger model using Crowley–Martin functional response

**DOI:** 10.1038/s41598-025-16107-0

**Published:** 2025-08-29

**Authors:** Rajalakshmi Manoharan, Reenu Rani, Manpreet Kaur, Anuj Kumar, Ali Moussaoui

**Affiliations:** 1 Department of Mathematics, School of Advanced Sciences, Vellore Institute of Technology, Vellore, Tamil Nadu 632014 India; 2 Department of Mathematics, Chandigarh Group of Colleges, Mohali, Punjab 140307 India; 3 School of Mathematics, Thapar Institute of Engineering and Technology, Patiala, Punjab 147004 India; 4 Laboratory of Nonlinear Analysis and Applied Mathematics, Department of Mathematics, University of Tlemcen, 13000 Tlemcen, Algeria

**Keywords:** Prey–predator–scavenger model, Local stability, Hopf bifurcations, Period-doubling, Chaos, Optimal harvesting policy, Numerical Simulations, Applied mathematics, Mathematics and computing

## Abstract

In the present article, a prey–predator–scavenger model is proposed and investigated with quadratic harvesting of predator and scavenger populations. The system is assumed to follow the Crowley–Martin functional response to describe the interaction between prey and predator populations. The positivity and boundedness of the system with respect to positive initial conditions are established. The analysis included determining all feasible equilibrium points and assessing their local stability under appropriate conditions. The system exhibits limit cycles around the interior equilibrium point. It is also observed that the solution of the system undergoes a period-doubling route to chaos. The existence of local bifurcation around the equilibrium points is investigated. It is shown that the system admits a transcritical bifurcation and a Hopf point for certain parameter values. The system also undergoes a global bifurcation, i.e., a generalized Hopf bifurcation, with respect to different parametric planes. The uniform persistence of the system is derived under specific conditions. Furthermore, an optimal harvesting problem is proposed and analyzed to determine the optimal harvesting pathways that not only maximize net revenue but also effectively manage harvesting efforts. The existence and characterization of optimal controls are discussed using Pontryagin’s maximum principle to balance the implementation of harvesting efforts. Extensive numerical simulations, including time series, phase portraits, and bifurcation diagrams, are performed to illustrate the theoretical results.

## Introduction

How do ecosystems maintain stability despite constant species interactions and environmental changes? Mathematical models helps to answer this question by providing a framework to study population dynamics, equilibrium states, and long-term patterns with the Lotka–Volterra^1,2^ system, stands out as a foundational model for numerous extensions. In particular, the predator-dependent functional response, such as Crowley–Martin functional response have been introduced to account for predator interference by assuming that the predator’s feeding rate is decreases when predator density is high, regardless of prey abundance, thereby providing a more realistic representation of prey–predator dynamics; see^3–6^.

Building on this, researchers have further expanded prey–predator models by introducing a third species to make the system more realistic, as it can significantly influence ecosystem dynamics. This additional species may play various roles, such as a competitor, an omnivore, or a scavengers, each contributing uniquely to population stability and species interactions. In this model, a scavenger is considered as a third species. Although scavengers are prevalent in nature, their influence in population dynamics has been explored in several studies. However, many aspects of their ecological role still require thorough examination. In this model, it is assumed that the scavenger species plays a dual role: (1) it is not purely dependent on dead animals but also acts as a predator of prey (since it actively hunts prey, it has negative impact on prey population dynamics); (2) it feeds on the dead predators, but this does not affect the predator population, as it only consumes animals that have already died naturally. This process helps to regulate decomposition and supports a healthy environment^7–12^. A real-world example that supports this interaction can be observed in marine ecosystems involving *Buccinum undatum* (common whelk)^7^, which functions as both a predator and scavenger. Whelks actively prey on live bivalves (they drill holes into the shells extract the soft tissue), while also scavenging on dead fish, including species like cod that are predators for bivalves. In this interaction, whelks do not attack or compete with live fish, and thus exert no negative impact on the predator population. Authors of^8^ examined a prey–predator–scavenger model employing a Michaelis–Menten type harvesting function. Additionally, Authors of^9^ studied a prey–predator model incorporating scavenger population and discovered that their model revealing complex behaviors such as Hopf bifurcations, bi-stability, and chaotic behavior. However, the effects of harvesting on such systems, particularly in the presence of scavengers, require further investigation.

Harvesting is a key factor in shaping the dynamics of ecological resources, as it directly affects the harvested natural population size and its sustainability. Therefore, understanding the effects of harvesting on multi-species ecosystems is crucial^13,14^, not only from an ecological perspective but also in terms of its economic consequences. Various types of harvesting functions have been proposed in the literature, such as linear, nonlinear, and quadratic harvesting. Numerous studies have analyzed the effects of linear and nonlinear harvesting functions^15–18^. Among these, studies show that nonlinear harvesting functions describe real-world situations better than linear ones, even though linear harvesting functions are still commonly used. Unlike linear harvesting, the Michaelis–Menten function^19^ introduces nonlinearity in the control variable. However, it still has certain limitations, so researcher of^20^ proposed a quadratic harvesting functions, which improves both biological and economic outcomes; see^7,11,12,21^.

The optimal harvesting control problem for a prey–predator–scavenger system focuses on determining the most effective strategies for managing populations in a three-species ecological model. The aim is to optimize harvesting across these interconnected species to achieve long-term benefits, such as economic profits, while ensuring ecosystem stability and sustainability^22^. This problem is modeled using differential equations involves control variables that represent the harvesting rates for each species^23–25^. The main challenge lies in navigating the complex interactions among these three-species populations while accounting for factors such as species growth rates, competition and environmental conditions. Kar et al.^26^ investigated the optimal control theory to obtain best strategies for the conservation of two-species system. Hacini et al.^27^ studied a predator-prey model employing an optimal harvesting policy designed to prevent species extinction, where the effort rate was used as a control variable.

In this paper, a prey–predator–scavenger system using the Crowley–Martin functional response is studied. The predator and scavenger population are subjected to quadratic harvesting. The study observes that the system exhibits a limit cycle around the interior equilibrium points. Additionally, it is noted that the system’s solution undergoing period-doubling may result in chaos. Finally, the possibility of an optimal control problem is investigated to maximize the net revenue with minimizing efforts.

The paper is structured in the following manner: in next section, the study commenced by examining the prey–predator–scavenger model incorporating quadratic harvesting. Subsequently, the positivity and boundedness of solutions are investigated in section “Preliminaries”. Sections “Existence of positive equilibria” and “Stability analysis of steady states” provides a concise overview of the existence and local stability of various equilibrium points, respectively. The local bifurcation of steady states (transcritical and Hopf bifurcation) is analyzed in section “Local bifurcation analysis” and the persistence conditions is derived in section “Uniform persistence”. The presence of stable, periodic solution, period-doubling and chaos solutions are investigated in numerical section “Numerical results”. Moreover, the existence of optimal controls and their numerical results are examined in section “Optimal control problem”. The concluding section summarizes the study’s findings.

## Model formulation

Let $X_1(T), X_2(T)$ and $X_3(T)$ denote the densities of prey, predator, and scavenger population, respectively, at time *T*. The model assumes the Lotka–Voltera prey–predator model with a Crowley–Martin functional response, described as follows:

1 $$\begin{aligned} \dfrac{dX_1}{dT}&= X_1(r-k X_1)-\dfrac{a X_1 X_2}{1+k_1 X_1+k_2 X_2+ k_3 X_1 X_2}, \nonumber \\ \dfrac{dX_2}{dT}&= \dfrac{c X_1 X_2}{1+ k_1 X_1+k_2 X_2+ k_3 X_1 X_2}-d X_2. \end{aligned}$$

**Fig. 1:**
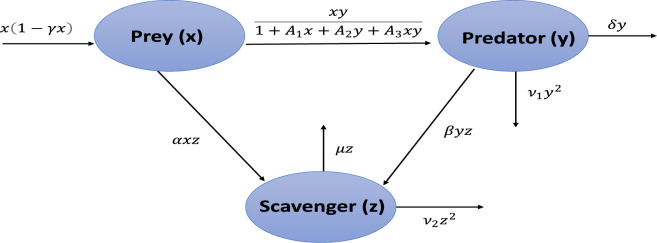
Schematic view for prey–predator–scavenger model.

The prey–predator–scavenger model can be formulated under the assumption that the scavenger population $(X_3)$ is predator for prey population $(X_1)$ and feeds on the natural carcasses of the predator population $(X_2)$, but this interaction does not impose any negative impact on the predator population. Schematic view for prey–predator–scavenger model is presented in Fig. 1. The defined model (1) and dynamics of scavenger population using quadratic harvesting of predator and scavenger populations is governed by the subsequent system of ordinary differential equations:

2 $$\begin{aligned} \dfrac{dX_1}{dT}&= X_1(r-k X_1)-\dfrac{a X_1 X_2}{1+k_1 X_1+k_2 X_2+ k_3 X_1 X_2}-bX_1 X_3, \nonumber \\ \dfrac{dX_2}{dT}&= \dfrac{c X_1 X_2}{1+ k_1 X_1+k_2 X_2+ k_3 X_1 X_2}-d X_2- q_1 E_1 X_2 ^2, \nonumber \\ \dfrac{dX_3}{dT}&= l X_1 X_3 +m X_2X_3 -n X_3 -q_2 E_2 X_3 ^2. \end{aligned}$$

Non-dimensionalizing the model (2) using the transformations $x = \dfrac{c}{r} X_1,\quad y = \dfrac{a}{r} X_2,\quad z=\dfrac{b}{r} X_3,\quad t = r T$ with dimensionless parameters^9^ described in Table 1.

The model is defined by the coupled dynamical equations, which are accompanied by the following initial conditions:

3 $$\begin{aligned} \frac{dx}{dt}&= x(1-\gamma x)-\dfrac{ xy}{1+ A_1 x+ A_2 y+ A_3 xy}-xz= xf_1(x,y,z),\nonumber \\ \frac{dy}{dt}&= \dfrac{x y}{1+A_1 x+A_2 y+ A_3 xy}-\delta y-\nu _1 y^2= yg_1(x,y,z), \nonumber \\ \frac{dz}{dt}&= \alpha x z+\beta y z-\mu z-\nu _2 z^2=zh_1(x,y,z). \end{aligned}$$

satisfying initial conditions

4 $$\begin{aligned} x(0)>0,\quad y(0)>0, \quad \text {and}\quad z(0)>0. \end{aligned}$$

The biological interpretation of all parameters in models (1), (2) and (3) is outlined in Table 1, with each parameters having non-negative numerical values.

**Table 1:** A biological description of all parameters included in systems (1), (2) and (3).

Parameters	Ecological definition	Non-dimensionlized representation
*r*	Intrinsic growth rate of prey population	
*r*/*k*	Carrying capacity of prey population	$\gamma =\dfrac{k}{c}$
$k_1$	Handling time on prey population	$A_1=\dfrac{k_1r}{c}$
$k_2$	Interference among predator population	$A_2=\dfrac{k_2r}{a}$
$k_3$	Interference among prey, predator population	$A_3=\dfrac{k_3r^2}{ca}$
*a*	Maximum attack rates of prey with respect to predator population	
*c*	Conversion rate to the predator’s growth	
*d*	Mortality rate of predator in the non-existence of prey	$\delta =\dfrac{d}{r}$
*b*	Maximum attack rates of prey with respect to scavenger	
$q_1,E_1$	Catchability and harvesting rate of predator population	$\nu _1=\dfrac{q_1E_1}{b}$
$q_2,E_2$	Catchability and harvesting rate of scavenger population	$\nu _2=\dfrac{q_2E_2}{a}$
*l*	Benefit rates to the scavenger’s growth from prey killed by scavenger population	$\alpha =\dfrac{l}{c}$
*m*	Benefit rates to the scavenger’s growth from died predator population	$\beta =\dfrac{m}{a}$
*n*	Mortality rate of scavenger in the non-existence of prey and predator population	$\mu =\dfrac{n}{r}$

## Preliminaries

This section establishes the positivity and boundedness of the system (3). Given that the variables *x*, *y*, and *z* represent population sizes, positivity guarantees that they remain non-negative. Boundedness indicates the natural limitation on growth imposed by finite resources.

### Positive invariance

#### Theorem 1

*All the solutions* (*x*(*t*), *y*(*t*), *z*(*t*)) *of the system* (3) *with the initial conditions are positive for all* $t>0$ .

#### Proof

The following solutions are obtained from each equation of the system (3).

5 $$\begin{aligned} x(t)&= x(0) exp\left( {\int _{0}^{t}f_1(x,y,z)dt}\right)>0,\nonumber \\ y(t)&= y(0) exp\left( {\int _{0}^{t}g_1(x,y,z)dt}\right)>0, \nonumber \\ z(t)&= z(0) exp\left( {\int _{0}^{t}h_1(x,y,z)dt}\right)>0, \quad \text {for all} \quad t>0. \end{aligned}$$

From Eq. (5) it can be verified that all the solutions of the system (3) with the initial conditions $x(0)>0, y(0)>0,$ and $z(0)>0$ are positive. Therefore, it can say that any solution from first quadrant of *xyz*-space gives a positive solution. $\square$

### Boundedness of the system

#### Theorem 2

*All the solutions of the system* (3) *which start in* $\textbf {R}^3$ *are uniformly bounded by the region*

$$\begin{aligned} Q&= \bigg \{(x,y,z)\in \textbf {R}^3; 0<h(t) \le M_1; \quad M_1=\dfrac{(1+M)^2}{4M\gamma }+\phi , \text { for any }\phi >0 \bigg \}, \quad \text {provided,}\\ &\quad \beta ^2<4\alpha \nu _1\nu _2 \end{aligned}$$

#### Proof

Let define the function as follows

$$\begin{aligned} h&=x+y+\frac{1}{\alpha }z\\ \dfrac{dh}{dt}&=\dfrac{dx}{dt}+\dfrac{dy}{dt} +\frac{1}{\alpha }\dfrac{dz}{dt} \\&=x(1-\gamma x)-\dfrac{ xy}{1+A_1 x+A_2 y+A_3 xy}-xz+\dfrac{x y}{1+A_1 x+A_2 y+A_3 xy}-\delta y-\nu _1 y^2\\&\quad +\frac{1}{\alpha } (\alpha x z+\beta y z-\mu z-\nu _2 z^2)\\&= x(1-\gamma x)-\delta y -\nu _1 y^2+ \frac{\beta y z}{\alpha } -\frac{\mu z}{\alpha }-\frac{\nu _2 z^2}{\alpha } \end{aligned}$$

After introducing a positive constant $M>0$, in a manner that $M=min\{\delta ,\mu \}$

6 $$\begin{aligned} \frac{dh}{dt}+Mh&=(1+M)x-\gamma x^2-(\delta -M)y-\frac{1}{\alpha }(\mu -M)z-\bigg (\nu _1 y^2-\frac{\beta }{\alpha }yz+\frac{\nu _2}{\alpha }z^2 \bigg ) \nonumber \\&\le (1+M)x-\gamma x^2-\bigg (\nu _1 y^2-\frac{\beta }{\alpha }yz+\frac{\nu _2}{\alpha }z^2 \bigg ) \end{aligned}$$

The term $\bigg (\nu _1 y^2-\dfrac{\beta }{\alpha }yz+\dfrac{\nu _2}{\alpha }z^2 \bigg )$ in the above equation is a quadratic form and can be seen to be positive definite for the condition $\beta ^2<4 \alpha \nu _1 \nu _2$. Therefore, the Eq. (6) reduces to

7 $$\begin{aligned} \frac{dh}{dt}+Mh\le (1+M)x-\gamma x^2 \end{aligned}$$

8 $$\begin{aligned} \frac{dh}{dt}+Mh\le \dfrac{(1+M)^2}{4\gamma } \end{aligned}$$

By using the theory of differential inequality, we get

$$\begin{aligned} 0<h(t)<\dfrac{(1+M)^2}{4M\gamma }(1-e^{-Mt})+h(0)e^{-Mt} \end{aligned}$$

And for the limit $t\rightarrow \infty$

9 $$\begin{aligned} 0<h(t)<\dfrac{(1+M)^2}{4M\gamma }=M_1 \end{aligned}$$

Hence, under the condition $\beta ^2<4\alpha \nu _1\nu _2$, all solutions of the system (3) with all positive initial conditions are constrained to the region

$$\begin{aligned} Q=\bigg \{(x,y,z)\in \textbf {R}^3; 0<x(t)+y(t)+\frac{1}{\alpha }z(t)=h(t) \le M_1; \quad M_1=\dfrac{(1+M)^2}{4M\gamma }+\phi , \text { for any }\phi >0 \bigg \}. \end{aligned}$$

$$\square$$

## Existence of positive equilibria

In this section, we are focused on all possible non-negative equilibrium points.

### Equilibrium points

(i) The trivial equilibrium point $P_0(0,0,0)$ is always exists.
(ii) Boundary equilibrium point $P_1\bigg (\dfrac{1}{\gamma },0,0\bigg ).$
(iii) Planar equilibrium point $P_2({\bar{x}},{\bar{y}},0)$ is a scavenger-free equilibrium point; ${\bar{x}}$ and ${\bar{y}}$ are the positive solutions of the equation given by
  10 $$\begin{aligned} (1-\gamma {\bar{x}})-\dfrac{{\bar{y}}}{1+ A_1 {\bar{x}}+ A_2 {\bar{y}}+ A_3 {\bar{x}}{\bar{y}}}=0 \end{aligned}$$
  11 $$\begin{aligned} \dfrac{{\bar{x}} }{1+A_1 {\bar{x}}+A_2 {\bar{y}}+ A_3 {\bar{x}}{\bar{y}}}-\delta -\nu _1 {\bar{y}}=0 \end{aligned}$$
(iv) Planar equilibrium point $P_3({\hat{x}},0,{\hat{z}})$ is the predator-free equilibrium point on *xz*-plane. where,
  12 $$\begin{aligned} {\hat{x}}=\dfrac{\mu +\nu _2}{\nu _2 \gamma +\alpha } \quad \text {and} \quad {\hat{z}}=\dfrac{\alpha -\gamma \mu }{\nu _2 \gamma +\alpha } \end{aligned}$$
  This equilibrium point exists when the following condition is satisfied.
  13 $$\begin{aligned} \alpha >\gamma \mu \end{aligned}$$
  This interprets that the benefit the scavenger gets from eating prey must be greater than the effect of its natural death rate, especially when the prey population is limited by the environment.
(v) $P^*(x^*, y^*,z^*)$ represents the interior equilibrium point of the system (3), with the solutions $x^*,y^*$, and $z^*$ being found by solving the following equations:
  14 $$\begin{aligned} (1-\gamma x^*)-\dfrac{y^*}{1+A_1 x^*+A_2 y^*+A_3 x^* y^*}-z^*&= 0, \nonumber \\ \dfrac{x^*}{1+A_1 x^*+A_2 y^*+A_3 x^*y^*}-\delta -\nu _1 y^*&= 0, \nonumber \\ \alpha x^*+\beta y^* -\mu -\nu _2 z^*&= 0. \end{aligned}$$
  Since the system includes nonlinear terms, finding a general algebraic solution may not always be feasible. However, numerical methods can be used to obtain the solutions $x^*,y^*$, and $z^*$ for chosen parameter values.

## Stability analysis of steady states

To evaluate the local stability of the system (3), the Jacobian matrix is determined at any point (*x*, *y*, *z*), is expressed as:

$$\begin{aligned} J= \left[ \begin{array}{ccc} x\left( -\gamma + \dfrac{y (A_1+A_3 y)}{(1+A_1 x+A_2 y+A_3 xy)^2} \right) +f_1 & \dfrac{-x(1+A_1 x)}{(1+A_1 x+A_2 y+A_3 xy)^2} & -x \\ \dfrac{y(1+A_2 y)}{(1+A_1 x+A_2 y+A_3 xy)^2} & y\left( \dfrac{-x(A_2+A_3 x)}{(1+A_1 x+A_2 y+A_3 xy)^2}- \nu _1 \right) +g_1 & 0 \\ \alpha z & \beta z & -\nu _2 z+h_1 \end{array} \right] \end{aligned}$$

### Stability of the equilibrium point $P_0$

The Jacobian matrix *J* is calculated at the point (0, 0, 0) for the system (3) is shown below:

$$\begin{aligned} J(0,0,0)= \left[ \begin{array}{ccc} 1 & 0 & 0\\ 0 & -\delta & 0\\ 0 & 0 & -\mu \end{array} \right] \end{aligned}$$

The determinantal equation of $J(P_0)$ is following

15 $$\begin{aligned} (\lambda -1)(\lambda + \delta ) (\lambda + \mu ) =0 \end{aligned}$$

The eigenvalues of Eq. (15) are $1,-\delta , \text {and} -\mu$. Therefore, the point $P_0$ is classified as a saddle point, since one eigenvalue is positive while the others are negative. This indicates that the origin (0, 0, 0) is dynamically unstable in the *x*-direction due to the positive eigenvalue, leading to an unstable manifold along that *x*-axis. The negative eigenvalues in the *y*- and *z*- directions correspond to stable manifolds. This interprets the existence of prey population.

### Stability of the equilibrium point $P_1$

The Jacobian matrix of the system (3) is determined at $\bigg (\dfrac{1}{\gamma }, 0, 0 \bigg )$ and presented below

$$J\bigg (\dfrac{1}{\gamma }, 0, 0 \bigg )= \left[ \begin{array}{ccc} -1 & -\dfrac{1}{\gamma +A_1} & -\dfrac{1}{\gamma }\\ 0 & -\delta +\dfrac{1}{\gamma +A_1} & 0\\ 0 & 0 & -\mu +\dfrac{\alpha }{\gamma } \end{array} \right]$$

The determinantal equation of $J(P_1)$ is following

16 $$\begin{aligned} (\lambda +1)\bigg (\lambda + \delta -\dfrac{1}{\gamma +A_1}\bigg ) \bigg (\lambda + \mu -\dfrac{\alpha }{\gamma }\bigg ) =0. \end{aligned}$$

The eigenvalues of (16) are $-1,-\delta +\dfrac{1}{\gamma +A_1}$ and $-\mu +\dfrac{\alpha }{\gamma }$. As a result, the equilibrium point $\bigg (\dfrac{1}{\gamma }, 0, 0 \bigg )$ is L.A.S iff the following conditions hold,

17 $$\begin{aligned} \gamma>\dfrac{\alpha }{\mu } \quad \text {and} \quad \gamma >\dfrac{1}{\delta }-A_1 \end{aligned}$$

This stability criterion can be summarized as:

18 $$\begin{aligned} \gamma >min\left( \dfrac{\alpha }{\mu },\dfrac{1}{\delta }-A_1\right) \end{aligned}$$

If the condition (18) is not satisfied, $P_1$ loses its stability and transitions into a saddle point. Notably, when either

19 $$\begin{aligned} \gamma =\dfrac{\alpha }{\mu } \quad \text {or (and)} \quad \gamma =\dfrac{1}{\delta }-A_1, \end{aligned}$$

the system approaches a critical threshold where bifurcation may occur. At these critical values, the system undergoes qualitative changes in its behavior.

The predator population can persist along with prey population in the nonexistence of scavenger population for the following conditions.

20 $$\begin{aligned} \gamma <\dfrac{\alpha }{\mu } \quad \text {and} \quad \gamma >\dfrac{1}{\delta }-A_1 \end{aligned}$$

Next, the scavenger population can also survive along with prey population in the absence of predator population for the following conditions.

21 $$\begin{aligned} \gamma >\dfrac{\alpha }{\mu } \quad \text {and} \quad \gamma <\dfrac{1}{\delta }-A_1 \end{aligned}$$

### Stability of the equilibrium point $P_2$

The Jacobian matrix of the system (3) is demonstrated at $({\bar{x}},{\bar{y}},0)$, is expressed as

$$\begin{aligned} J({\bar{x}},{\bar{y}},0)= \left[ \begin{array}{ccc} a_{11}& a_{12} & -{\bar{x}} \\ a_{21} & a_{22} & 0 \\ 0 & 0 & a_{33} \end{array} \right] \end{aligned}$$

The determinantal equation of $J(P_2)$ is following

22 $$\begin{aligned} (\lambda -a_{33}) (\lambda ^2-\lambda (a_{11}+a_{22})+a_{11} a_{22}-a_{21} a_{12} )=0 \end{aligned}$$

$$\begin{aligned} a_{11}&= {\bar{x}}\left( -\gamma +\dfrac{{\bar{y}}(A_1+A_3{\bar{y}})}{(1+A_1 {\bar{x}}+A_2 {\bar{y}}+A_3 {\bar{x}}{\bar{y}})^2}\right) , \quad a_{12} = \dfrac{- {\bar{x}} (1+A_2 {\bar{x}})}{(1+A_1 {\bar{x}}+A_2 {\bar{y}}+A_3 {\bar{x}} {\bar{y}})^2}, \\ a_{21}&= \dfrac{{\bar{y}}\left( 1+A_2 {\bar{y}} \right) }{(1+A_1 {\bar{x}}+A_24 {\bar{y}}+A_3 {\bar{x}}{\bar{y}})^2}, a_{22} = {\bar{y}}\left( -\dfrac{{\bar{x}}(A_2+A_3{\bar{x}})}{(1+A_1 {\bar{x}}+A_2 {\bar{y}}+A_3 {\bar{x}}{\bar{y}})^2}-\nu _1\right) , \quad a_{33} = \alpha {\bar{x}}+ \beta {\bar{y}}-\mu . \end{aligned}$$

The Eq. (22) has a root $\lambda = a_{33}$, while the remaining two roots are determined by solving the following equation:

$$\begin{aligned} \lambda ^2-\lambda (a_{11}+a_{22})+a_{11} a_{22}-a_{21} a_{12}=0. \end{aligned}$$

Hence, the point $P_2$ is locally asymptotically stable when the conditions

23 $$\begin{aligned} T_1=a_{11}+a_{22}<0, \quad D_1=a_{11} a_{22}-a_{12} a_{21}>0, \quad \text { and } \quad \lambda =a_{33}<0 \end{aligned}$$

hold true. Conversely, if the conditions are not met, specifically if $a_{33}>0$, the equilibrium point becomes a saddle point, indicating instability along *z*-axis. This interprets the coexistence of all species.

Additionally, when $T_1<0$ and $D_1=0$, the system exhibits a transcritical bifurcation around $P_2$, leading to a change in the stability and system’s behavior, as explored in Theorem 3.

### Stability of the equilibrium point $P_3$

The following Jacobian matrix of the system (3) calculated at $({\hat{x}},0,{\hat{z}})$ and presented as follows

$$J({\hat{x}},0,{\hat{z}})= \left[ \begin{array}{ccc} b_{11} & b_{12} & b_{13}\\ 0 & b_{22} & 0\\ b_{31} & b_{32} & b_{33} \end{array} \right]$$

The determinantal equation of $J(P_3)$ is following

24 $$\begin{aligned} (\lambda -b_{22}) (\lambda ^2-\lambda (b_{11}+b_{33})+b_{11} b_{33}-b_{31} b_{13} )=0 . \end{aligned}$$

where, $$b_{11}=-\gamma {\hat{x}},$$ $$b_{12}=-\dfrac{{\hat{x}}}{1+A_1{\hat{x}}},$$ $$b_{13}=-{\hat{x}},$$ $$b_{22}=-\delta +\dfrac{{\hat{x}}}{1+A_1{\hat{x}}},$$ $$b_{31}=\alpha {\hat{z}},$$ $$b_{32}=\beta {\hat{z}},$$ $$b_{33}=-\nu _2 {\hat{z}}.$$

 One root of the Eq. (24) is $\lambda = b_{22}$ and the rest of two eigenvalues can be calculated from the following equation,

$$\begin{aligned} \lambda ^2-\lambda (b_{11}+b_{33})+b_{11} b_{33}-b_{31} b_{13}=0 \end{aligned}$$

Here, the trace and the determinant of the characteristic equation are as follows,

25 $$\begin{aligned} T_2=b_{11}+b_{33}=-\gamma {\hat{x}}-\nu _2{\hat{z}}<0, \quad D_2=b_{11} b_{33}-b_{31} b_{13} =(\gamma \nu _2+\alpha ){\hat{x}}{\hat{z}}>0. \end{aligned}$$

The condition (25) suggest that two of the characteristic roots have negative real parts. However, the overall stability of the point $P_3$ is determined by the eigenvalue $\lambda =b_{22}$. Therefore, the point $P_3$ is L.A.S if it is satisfies the subsequent condition:

26 $$\begin{aligned} \nu _2+\mu <\dfrac{\delta (\nu _2\gamma +\alpha )}{1-\delta A_1} \end{aligned}$$

The inequality ensures that the eigenvalue $b_{22}$ is also negative, leading to stability. Alternatively, if this condition (26) is violated, the stability is lost, and the point $P_3$ becomes a saddle point along the *y*- axis.

Biologically, we can say predator population also survive that gives the coexistence of all species for the following condition:

27 $$\begin{aligned} \nu _2+\mu >\dfrac{\delta (\nu _2\gamma +\alpha )}{1-\delta A_1} \end{aligned}$$

### Stability of the equilibrium point $P^*$

The determinantal equation of the Jacobian matrix $J(P^*)$ is stated below

28 $$\begin{aligned} \lambda ^3+\rho _1 \lambda ^2+\rho _2 \lambda +\rho _3=0. \end{aligned}$$

where

$$\begin{aligned} \rho _1&=-(c_{11}+c_{22}+c_{33}) \\ \rho _2&=c_{22}c_{33}+c_{11} c_{33}-c_{13}c_{31}+c_{11}c_{22}-c_{21}c_{12}\\ \rho _3&=c_{12}c_{21}c_{33}+c_{31}c_{22}c_{13}-c_{11}c_{22}c_{33}-c_{13}c_{21}c_{32} \end{aligned}$$

where, $$c_{11} = x^*\left( -\gamma +\dfrac{y^*(A_1+A_3y^*)}{(1+A_1 x^*+A_2 y^*+A_3 x^*y^*)^2}\right) , \quad c_{12} = -\dfrac{x^*(1+A_1 x^*)}{(1+A_1 x^*+A_2 y^*+A_3 x^*y^*)^2} ,$$ $$c_{13} = -x^*, \quad c_{21}= \dfrac{y^*(1+A_2 y^* )}{(1+A_1 x^*+A_2 y^*+A_3x^*y^*)^2}$$, $$c_{22}= y^*\left( -\dfrac{x^*(A_2+A_3x^*)}{(1+A_1 x^*+A_2 y^*+A_3 x^*y^*)^2}-\nu _1\right)$$, $$c_{31} = \alpha z^* , \quad c_{32} =\beta z^*, \quad c_{33}= -\nu _2 z^*$$.

According to Routh–Hurwitz stability criterion, $P^*(x^*,y^*,z^*)$ is L.A.S if $\rho _1>0$, $\rho _3>0$ and $\rho _1 \rho _2-\rho _3>0$. Hence, to demonstrate the stability of $P^*$ (interior point), a numerical example is used due to the complexity of the algebraic expression. Consider the set of parameter values $\gamma =0.9, A_1=0.11, A_2=0.13, A_3=0.4, \alpha =0.3, \beta =13,\nu _1=0.07$and $\nu _2=0.3$. The unique feasible coexistence equilibrium point $P^*=(0.3888,0.7992,0.0212)$ is obtained, and the Eq. (28) becomes,

$$\begin{aligned} \lambda ^3+(0.3846) \lambda ^2+(0.1716)\lambda +0.0599=0. \end{aligned}$$

Since $\rho _1>0$, $\rho _3>0$ and $\rho _1\rho _2-\rho _3=0.0061>0$ that confirms the point $P^*$ is locally asymptotically stable.

## Local bifurcation analysis

### Transcritical analysis

#### Theorem 3

*The system* (3) *around the planar equilibrium point* $P_2({\bar{x}},{\bar{y}},0)$ *at the bifurcation parameter *$\beta$ *exhibits a transcritical bifurcation if*

$$\begin{aligned} \beta =\beta ^{[TC]} \end{aligned}$$

#### Proof

The Jacobian matrix $J({\bar{x}},{\bar{y}},0)$ indicates that the system (3) at the planar equilibrium point $P_2$ has a zero eigenvalue, denoted as $\lambda =a_{33}=0$ when $\beta =\beta ^{[TC]}$. The Jacobian matrix $J^*(P_2)$ at this point becomes:

29 $$\begin{aligned} J^*(P_2,\beta ^{[TC]})= \begin{bmatrix} a_{11} & a_{12} & a_{13} \\ a_{21} & a_{22} & 0 \\ 0 & 0 & 0 \end{bmatrix} \end{aligned}$$

where, $$a_{11} = {\bar{x}}\left( -\gamma +\dfrac{{\bar{y}}(A_1+A_3{\bar{y}})}{B}\right) ,$$ $$a_{12} = \dfrac{-{\bar{x}}(1+A_1 {\bar{x}})}{B},$$ $$a_{13} = -{\bar{x}},$$ $$a_{21} = \dfrac{{\bar{y}}(1+A_2 {\bar{y}})}{B},$$ $$a_{22} = {\bar{y}}\left( -\dfrac{{\bar{x}}(A_2+A_3{\bar{x}})}{B}-\nu _1\right)$$

Where, $B=(1+A_1 {\bar{x}}+A_2 {\bar{y}}+A_3 {\bar{x}}{\bar{y}})^2$. Let $U=(u_1,u_2,1)^T$ and $W=(w_1,w_2,w_3)^T=(0,0,1)^T$ be the eigenvectors of $J^*(P_2)$ and $J^*(P_2)^T$ corresponding to the eigenvalue $\lambda =a_{33}=0$, respectively, which follows:

$$\begin{aligned} u_1&= - \dfrac{(B(A_3{\bar{x}}^2 + A_2{\bar{x}} + B\nu _1))}{(- A_3^2{\bar{x}}^2{\bar{y}}^2 + \gamma A_3B{\bar{x}}^2 - \nu _1A_3B{\bar{y}}^2 - A_1A_3{\bar{x}}^2{\bar{y}}- A_2A_3{\bar{x}}{\bar{y}}^2 + \gamma \nu _1B^2 + A_2\gamma B{\bar{x}} - A_1\nu _1B{\bar{y}} + A_1{\bar{x}} + A_2{\bar{y}} + 1)}\\ u_2&= -\dfrac{(B(A_2{\bar{y}} + 1))}{(- A_3^2{\bar{x}}^2{\bar{y}}^2 + \gamma A_3B{\bar{x}}^2 - \nu _1A_3B{\bar{y}}^2 - A_1A_3{\bar{x}}^2{\bar{y}} - A_2A_3{\bar{x}}{\bar{y}}^2 + \gamma \nu _1B^2 + A_2\gamma B{\bar{x}} - A_1\nu _1B{\bar{y}} + A_1{\bar{x}} + A_2{\bar{y}} + 1)} \end{aligned}$$

Let $F=(xf_1,yg_1,zh_1)^T$. The conditions of Sotomayar’s theorem can be checked as follows:

$$\begin{aligned} \Delta _1 = W^T F_{\beta }(P_2,\beta ^{[TC]}) = 0 \end{aligned}$$

Since $\Delta _1=0$, a saddle-node bifurcation around $P_2$ is ruled out. The computation of $\Delta _2$ is as follows:

$$\begin{aligned} \Delta _2&= W^T [D F_{\beta }(P_2,\beta ^{[TC]})U] = {\bar{y}}u_3 \quad \ne 0 \end{aligned}$$

here, $$D F_{\beta }(P_2,\beta ^{[TC]})=\begin{bmatrix} 0 & 0 & 0\\ 0 & 0 & 0\\ 0 & 0 & {\bar{y}} \end{bmatrix}$$ and

$$\begin{aligned} \Delta _3&= W^T [D^2 F_{\beta }(P_2,\beta ^{[TC]})(U,U)]\\&= -2 \nu _2 u_3^2+2\beta u_2 u_3+2\alpha u_1 u_3 \quad \ne 0 \end{aligned}$$

Hence, all conditions required by Sotomayar’s theorem for a transcritical bifurcation are satisfied. Thus, the system (3) admits a transcritical bifurcation around the point $P_2({\bar{x}},{\bar{y}},0)$. $\square$

For a particular data set (39), the Jacobian matrix *J* of $P_2(0.393409, 0.827479, 0)$ is confirmed to possess a zero eigenvalue at $\beta =12.546517$, (bifurcation parameter). This confirmation remains consistent under the specified parameters: $\gamma =0.9, A_1=0.11, A_2=0.13, A_3=0.4, \alpha =0.3, \nu _1=0.069$ and $\nu _2=0.3$. The Jacobian matrix $J^*(P_2,\beta ^{[TC]})$ is as follows,

30 $$\begin{aligned} J^*(P_2,\beta ^{[TC]})=\begin{bmatrix} -0.2666 & -0.2501 & -0.3934 \\ 0.5585 & -0.1141 & 0 \\ 0 & 0 & 0 \end{bmatrix} \end{aligned}$$

**Fig. 2:**
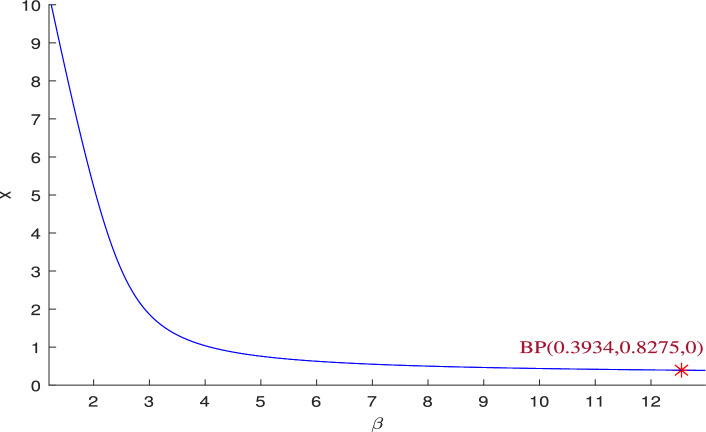
One parametric bifurcation diagram for the system (3) w.r.t the parameter $\beta$, which represents the benefit rates to the scavenger’s growth from naturally died predator population.

The eigenvector *U* corresponding to $\lambda =a_{33}=0$ is $(-0.1595,-0.7806,0.6043)^T$, and $W=(0,0,1)^T$. The analysis confirms $\Delta _1=0$ and $\Delta _2,\Delta _3$ as non-zero, satisfying the Sotomayor’s conditions. Therefore, the system (3) undergoes a transcritical bifurcation (BP) at $\beta$, as given in Fig. 2. This plot shows that the transcritical bifurcation will occur at the critical value $\beta =12.546517$ for the data set (39). At the bifurcation point $\beta =\beta ^{[TC]}$, the scavenger population transitions between extinction and persistence. If increasing the parameter $\beta$, increases the survival rate of the scavenger, then the scavenger species survives after the bifurcation threshold value due to sufficient availability of naturally dead predators. However, when $\beta <\beta ^{[TC]}$, the scavenger species tends toward extinction due to insufficient availability of predator carcasses to support its survival.

#### Remark 1

The system (3) also admits a transcritical bifurcation at $\nu _1$ around the point $P_2({\bar{x}},{\bar{y}},0)$ if

$$\begin{aligned} \nu _1=\nu _1^{[TC]} \end{aligned}$$

According to the Jacobian matrix $J({\bar{x}},{\bar{y}},0)$, the system (3) around the point $P_2$ has a zero eigenvalue say $\lambda =a_{33}=0$ at $\nu _1=\nu _1^{[TC]}$ and the Jacobian matrix $J^*(P_2,\nu _1^{[TC]})$ as given in (29). Similarly, it can be seen that $\Delta _1=0,\Delta _2\ne 0$ and $\Delta _3\ne 0$. Hence, the system (3) admits a transcritical bifurcation around $P_2({\bar{x}},{\bar{y}},0)$.

The data set (39) confirms the presence of a zero eigenvalue in Jacobian matrix $J({\bar{x}},{\bar{y}},0)$ with respect to bifurcation parameter $\nu _1=0.097250$ at $P_2(0.420748,0.797983,0)$ by selecting specific numerical values $r=0.9,A_1=0.11,A_2=0.13,A_3=0.4,\alpha =0.3,\beta =13,\mu =10.5$. Consequently, the Jacobian matrix $J^*(P_2)$ for the system (3) is expressed as,

31 $$\begin{aligned} J^*(P_2,\nu _1^{[TC]})=\begin{bmatrix} -0.2913 & -0.2669 & -0.4207\\ 0.5340 & -0.1383 & 0\\ 0 & 0 & 0 \end{bmatrix} \end{aligned}$$

**Fig. 3:**
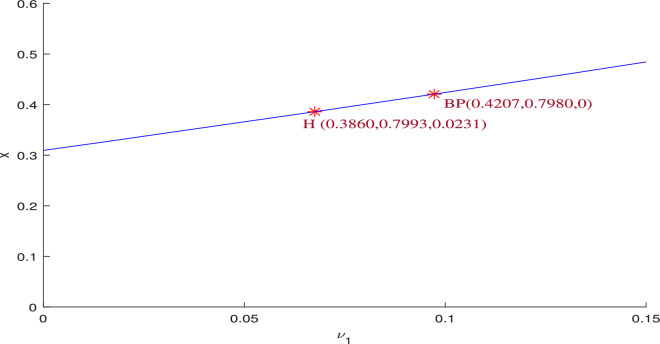
One parametric bifurcation diagram for the system (3) w.r.t $\nu _1$ for the data set (39). The Hopf bifurcation point takes place at the threshold value $\nu _1=0.0675$ with first Lyapunov coefficient being $-1.941017 e^{+01} (<0)$. This signifies a supercritical Hopf bifurcation, indicates the presence of a stable limit cycle. This diagram reveals that increasing the harvesting rate on the predator population initially leads to population oscillations among all three species for $0<\nu _1<\nu _1^H$. As $\nu _1$ increases further, the system stabilizes, allowing coexistence of prey, predator, and scavenger population for $\nu _1^H<\nu _1<\nu _1^{[TC]}$. However, when $\nu _1>\nu _1^{[TC]}$, the scavenger goes extinct due to excessive harvesting, which depletes the predator population and results in insufficient predator carcasses availability, which is critical for scavenger survival.

The eigenvector *U* corresponding to $\lambda =a_{33}=0$ is $(-0.1970,-0.7605,0.6188)^T$, and $W=(0,0,1)^T$. From this, it is straightforward to verify that $\Delta _1=0$, while $\Delta _2$ and $\Delta _3$ are non-zero, satisfying Sotomayor’s conditions. Thus, the system (3) admits a transcritical bifurcation (BP) at $\nu _1$, as displayed in Fig. 3.

### Hopf bifurcation analysis

Let’s examine the Hopf bifurcation for an interior equilibrium point with respect to the bifurcation parameter $\nu _1$. Determining the components of the equilibrium point $P^*$ is challenging due to algebraic complexity. However, its instability can be analyzed using the Hopf bifurcation criteria, specifically by evaluating the determinantal equation of the Jacobian matrix according to Liu’s criterion^28–30^.

**Liu’s criterion:** Let

32 $$\begin{aligned} \lambda ^3+\rho _1(\nu _1) \lambda ^2+\rho _2(\nu _1) \lambda +\rho _3(\nu _1)=0, \end{aligned}$$

be the determinantal equation of the Jacobian matrix at an interior equilibrium point, where $\rho _1(\nu _1), \rho _2(\nu _1)$ and $\rho _3(\nu _1)$ are the smooth function of $\nu _1$ such that the following conditions hold for some threshold value $\nu _1^H$,

1. $$\rho _1(\nu _1^H)>0, \quad \rho _3(\nu _1^H)>0,$$    $$\phi (\nu _1^H)=\rho _1(\nu _1^H)\rho _2(\nu _1^H)-\rho _3(\nu _1^H)=0$$
2. $$\dfrac{d\phi (\nu _1^H)}{d\nu _1^H}\ne 0$$

where,

$$\begin{aligned} \phi (\nu _1^H)&= (\nu _2 z+x(\gamma -(y(A_1+A_3y))/(A_1 x+A_2 y+A_3 x y+1)^2)+y(\nu _1+(x(A_2+A_3x))/\\ &\quad (A_1 x+A_2 y+A_3 x y+1)^2))(\alpha x z+xy(\gamma -(y(A_1+A_3y))/(A_1 x+A_2 y+A_3 x y+1)^2)\\ &\quad (\nu _1+(x(A_2+A_3x))/(A_1 x+A_2 y+A_3 x y+1)^2)+\nu _2 xz(\gamma -(y(A_1+A_3y))\\ &\quad /(A_1 x+A_2 y+A_3 x y+1)^2)+\nu _2 yz(\nu _1+(x(A_2+A_3x))/(A_1 x+A_2 y+A_3 x y+1)^2)\\ &\quad +(xy(A_1x+1)(A_2y+1))/(A_1 x+A_2 y+A_3 x y+1)^4)-\alpha x y z(\nu _1+(x(A_2+A_3x))\\ &\quad /(A_1 x+A_2 y+A_3 x y+1)^2)-\nu _2 x y z(\gamma -(y(A_1+A_3y))/(A_1 x+A_2 y+A_3 x y+1)^2)\\ &\quad (\nu _1+(x(A_2+A_3x))/(A_1 x+A_2 y+A_3 x y+1)^2)-(\beta x y z(A_2 y+1))/(A_1 x+A_2 y+A_3 x y+1)^2\\ &\quad -(\nu _2 x y z(A_1 x+1)(A_2y+1))/(A_1 x+A_2 y+A_3 x y+1)^4\\ \dfrac{d\phi (\nu _1^H)}{d\nu _1^H}&= y(\alpha x z+x y(\gamma -(y(A_1+A_3y))/((A_1 x+A_2 y+A_3 x y+1)^2)(\nu _1+(x(A_2+A_3x))\\ &\quad /(A_1 x+A_2 y+A_3 x y+1)^2)+\nu _2xz(\gamma -(y(A_1+A_3y))/(A_1 x+A_2 y+A_3 x y+1)^2)\\ &\quad +\nu _2yz(\nu _1+(x(A_2+A_3x))/(A_1 x+A_2 y+A_3 x y+1)^2)+(xy(A_1x+1)(A_2y+1))\\ &\quad /(A_1 x+A_2 y+A_3 x y+1)^4)+(xy(\gamma -(y(A_1+A_3y))/(A_1 x+A_2 y+A_3 x y+1)^2)+\nu _2yz)\\ &\quad (\nu _2z+x(\gamma -(y(A_1+A_3y))/(A_1 x+A_2 y+A_3 x y+1)^2)+y(\nu _1+(x(A_2+A_3x))/\\ &\quad (A_1 x+A_2 y+A_3 x y+1)^2))-\alpha x y z-\nu _2xyz(\gamma -(y(A_1+A_3y))/(A_1 x+A_2 y+A_3 x y+1)^2) \end{aligned}$$

The parameter values are fixed as given in (39), revealing a Hopf bifurcation threshold at $\nu _1^H=0.0675$ for the point $P_2(0.3860,0.7993,0.0231)$. The conditions of the Liu’s criterion at $\nu _1^H$ are following:

$$\begin{aligned} & \rho _1(\nu _1^H)= 0.3805>0, \quad \rho _3(\nu _1^H)=0.0649>0,\\ & \phi (\nu _1^H)=\rho _1(\nu _1^H)\rho _2(\nu _1^H)-\rho _3(\nu _1^H)=0, \quad \dfrac{d(\phi (\nu _1^H))}{d\nu _1^H} =0.2155\ne 0. \end{aligned}$$

Thus, Hopf bifurcation occurs at $\nu _1=\nu _1^H$ (see in Fig. 3).

## Uniform persistence

In ecology, the term “persistence” describes the continued survival of all species in a system over an extended period. It means that all solutions at the equilibrium point should be unstable at the boundary axes but may be either stable or form a limit cycle within the interior. The persistence of the system (3) is demonstrated using the well-known technique of the “average Lyapunov function”^7,31^.

**Fig. 4:**
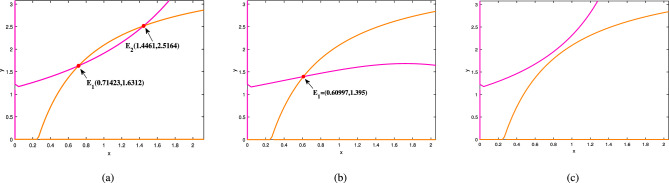
For the parameter set (39), (**a**) shows the presence of two equilibrium points at $\gamma =0.1$. In (**b**), it is shown that as $\gamma$ increases to 0.2, the two equilibrium points collides, resulting in one equilibrium point. Lastly, in (**c**), there is no equilibrium points exist for $\gamma =0.01$.

**Fig. 5:**
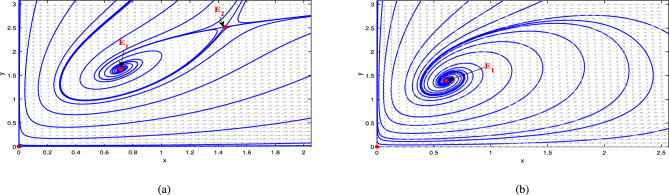
Dynamical behavior of the system (33) w.r.t time *t* is given for (**a**) $\gamma =0.1$, and (**b**) $\gamma =0.2$.

**Fig. 6:**
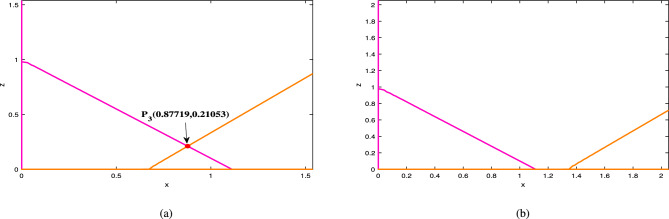
For the parameter set (39), (**a**) shows a single equilibrium point at $\mu =0.2$, and in (**b**), no equilibrium points exist for $\mu =0.4$.

**Fig. 7:**
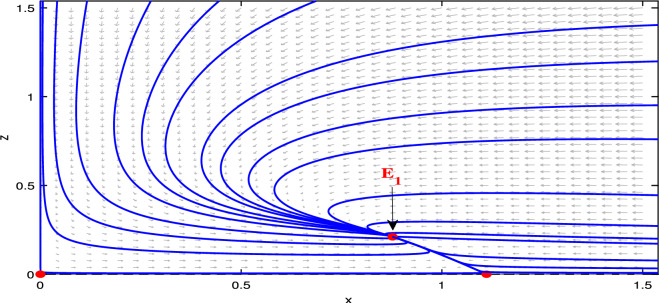
Dynamical behavior of the system (34) w.r.t time *t* is given for $\mu =0.2$.

Before, studying the persistence of the system (3) , the global dynamics on the boundary planes *xy* and *xz* are need to be discussed. It can be seen that the system (3) consists of two subsystem where subsystem-I (33) and subsystem-II (34) are given as follows:

Moreover, the existence of equilibrium and dynamical behavior of the subsystems-I and II are obtained and illustrated in Figs. 4, 5, 6 and 7.

### Subsystem-I

33 $$\begin{aligned} \frac{dx}{dt}&= x(1-\gamma x)-\dfrac{ xy}{1+ A_1 x+ A_2 y+ A_3 xy}=f_{11}(x,y), \nonumber \\ \frac{dy}{dt}&= \dfrac{x y}{1+A_1 x+A_2 y+ A_3 xy}-\delta y-\nu _1 y^2=g_{11}(x,y). \end{aligned}$$

### Subsystem-II

34 $$\begin{aligned} \frac{dx}{dt}&= x(1-\gamma x)-xz=f_{12}(x,z), \nonumber \\ \frac{dz}{dt}&= \alpha x z-\mu z-\nu _2 z^2=h_{11}(x,z). \end{aligned}$$

#### Theorem 4

*There does not exist non-trivial periodic dynamics inside the positive quadrant of* *xy*-*plane*.

#### Proof

The following form describes a continuous differential function within the interior of *xy*-plane,

$$\begin{aligned} D(x,y)=\frac{1}{xy}, (\text {Dulac function}) \end{aligned}$$

The Bendixson–Dulac criterion for the subsystem (33) is presented below:

$$\begin{aligned} B_{D}(x,y)&= \dfrac{\partial (Df_{11})}{\partial x}+\dfrac{\partial (Dg_{11})}{\partial y}\\&= -\frac{\gamma }{y}-\frac{\nu _1}{x}+\dfrac{A_1+A_3y}{B}-\dfrac{(A_2+A_3x)}{B} \end{aligned}$$

Clearly $B_{D}(x,y)$ is not identically zero and maintains a consistent sign within the interior of the positive quadrant of *xy* -plane, provided the following condition is met :

35 $$\begin{aligned} \dfrac{A_1+A_3y}{B}<\frac{\gamma }{y}+\frac{\nu _1}{x}+\dfrac{(A_2+A_3x)}{B} \end{aligned}$$

Thus, there are no non-trivial periodic dynamics inside the positive quadrant of *xy* - plane.

Similarly, the subsystem (34) shows there is no non-trivial periodic dynamics within the positive quadrant of *xz*-plane provided

36 $$\begin{aligned} \frac{\gamma }{z}+\frac{\nu _2}{x}>0 \end{aligned}$$

$$\square$$

#### Theorem 5

*The system* (3) *is uniformly persistent provided*

37 $$\begin{aligned} &\text {(i)}\qquad \gamma<\dfrac{\alpha }{\mu }\quad \text {and} \quad \gamma <\dfrac{1}{\delta }-A_1 \nonumber \\ &\text {(ii)}\qquad \alpha {\bar{x}}+\beta {\bar{y}}> \mu \nonumber \\ &\text {(iii)}\qquad \nu _2+\mu> \dfrac{\delta (\nu _2\gamma +\alpha )}{1-\delta A_1} \end{aligned}$$

#### Proof

Consider the average Lyapunov function for (*x*, *y*, *z*) as follows,

38 $$\begin{aligned} L(x,y,z)=x^{m_1} y^{m_2} z^{m_3} \end{aligned}$$

where $m_1,m_2$ and $m_3$ are positive constants. Differentiating (38) using the expression of time derivative of *x*, *y* and *z* from (3). which follows,

$$\begin{aligned} \dfrac{{\dot{L}}}{L}& = \kappa (x,y,z)=m_1 \dfrac{{\dot{x}}}{x}+m_2 \dfrac{{\dot{y}}}{y}+m_3 \dfrac{{\dot{z}}}{z}\\&= m_1\left( 1-\gamma x-\dfrac{ y}{1+ A_1 x+ A_2 y+ A_3 xy}-z\right) +m_2\left( \dfrac{ y}{1+A_1 x+A_2 y+ A_3 xy}-\delta -\nu _1 y\right) \\ &\quad +m_3\left( \alpha x +\beta y -\mu -\nu _2 z\right) \end{aligned}$$

The system is considered uniformly persistence if $\kappa (x,y,z)>0$ at all equilibrium points $P_i, (i=0,1,2,3)$ and are computed as follows:

$$\begin{aligned} P_0:\kappa (0,0,0)&= m_1-m_2\delta -m_3\mu \\ P_1:\kappa \bigg (\dfrac{1}{\gamma },0,0\bigg )&= m_2\left( \dfrac{1}{\gamma +A_1}-\delta \right) +m_3\left( \dfrac{\alpha -\gamma \mu }{\gamma }\right) \\ P_2:\kappa ({\bar{x}},{\bar{y}},0)&= m_1\left( 1-\gamma {\bar{x}}-\dfrac{{\bar{y}}}{1+A_1{\bar{x}}+A_2{\bar{y}}+A_3{\bar{x}}{\bar{y}}}\right) +m_2\left( \dfrac{{\bar{x}}}{1+A_1{\bar{x}}+A_2{\bar{y}}+A_3{\bar{x}}{\bar{y}}}-\delta -\nu _1{\bar{y}}\right) \\ &\quad m_3\left( \alpha {\bar{x}}+\beta {\bar{y}}-\mu \right) \\ P_3:\kappa ({\hat{x}},0,{\hat{z}})&= m_1\left( 1-\gamma {\hat{x}}-{\hat{z}}\right) +m_2\left( \dfrac{{\hat{x}}}{1+A_1{\hat{x}}}-\delta \right) +m_3\left( \alpha {\hat{x}}-\mu -\nu _2{\hat{z}}\right) \end{aligned}$$

Since $m_1>m_2\delta +m_3\mu$, $\kappa (0,0,0)$ is positive at equilibrium point $P_0$. Additionally, $\kappa \bigg (\dfrac{1}{\gamma },0,0\bigg )$ is positive provided

$$\begin{aligned} \gamma<\dfrac{\alpha }{\mu } \quad \text {and} \quad \gamma <\dfrac{1}{\delta }-A_1 \end{aligned}$$

Moreover, $\kappa ({\bar{x}},{\bar{y}},0)$ remains positive when the conditions $\alpha {\bar{x}}+\beta {\bar{y}}>\mu$ is satisfied. Similarly, $\kappa ({\hat{x}},0,{\hat{z}})$ is positive if

$$\begin{aligned} \nu _2+\mu >\dfrac{\delta (\nu _2\gamma +\alpha )}{1-\delta A_1} \end{aligned}$$

This implies that, under these constraints, the system (3) is uniformly persistent over time. $\square$

## Numerical results

Numerical results are presented for the following parametric choices to validate the analytical findings. This section also examines the dynamical behavior of the system (3) and the parametric values are listed below:

39 $$\begin{aligned} \gamma =0.9, A_1=0.11, A_2=0.13, A_3=0.4, \delta =0.25, \alpha =0.3, \beta =13, \mu =10.5, \nu _2=0.3 \end{aligned}$$

**Fig. 8:**
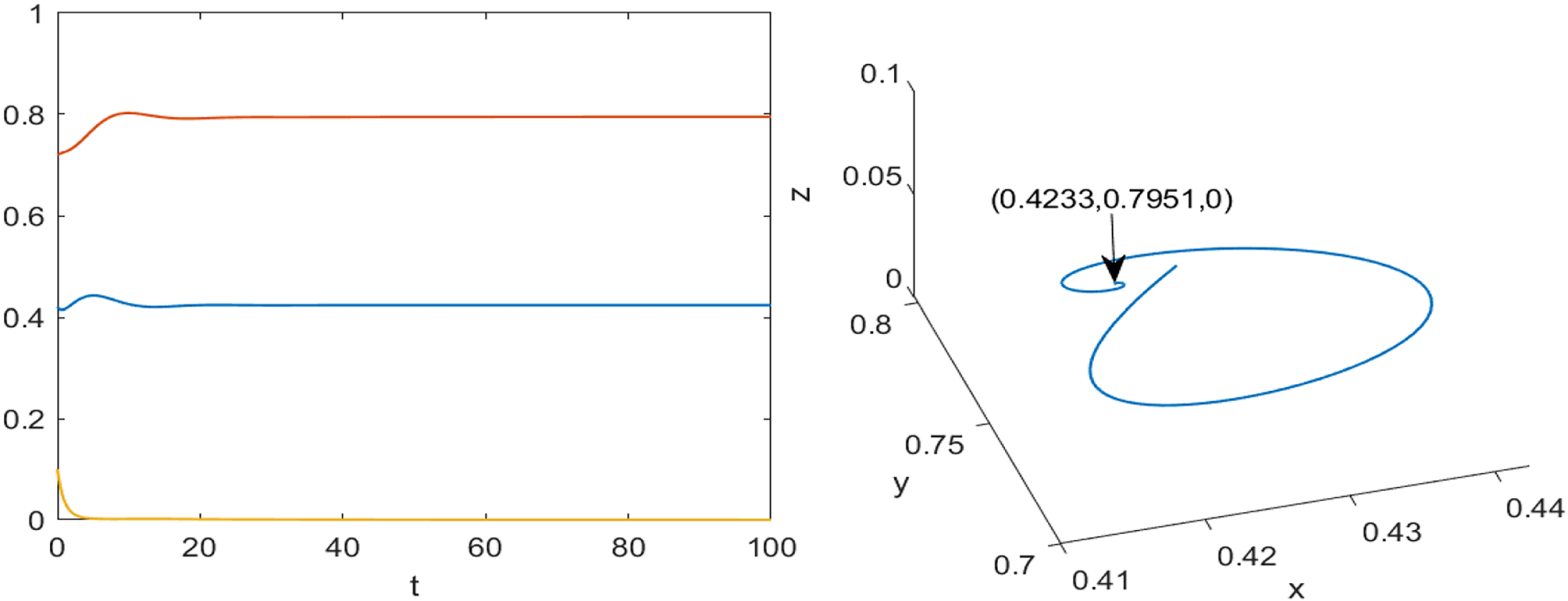
Time series and phase portrait for the planar point $P_2({\bar{x}},{\bar{y}},0)$ at $\nu _1=0.1$.

**Fig. 9:**
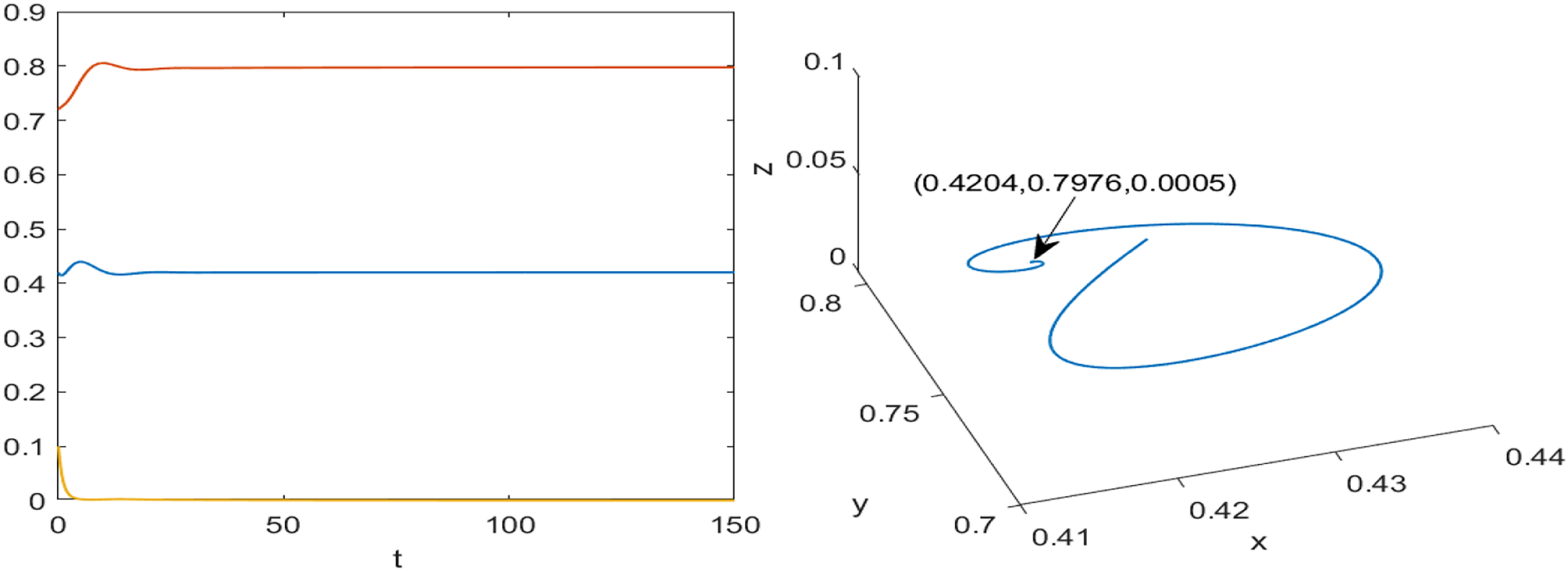
Time series and phase portrait of the system (3) for $\nu _1=0.097$ shows stable solutions in $\textbf {R}^3$ for the set of parameters (39).

**Fig. 10:**
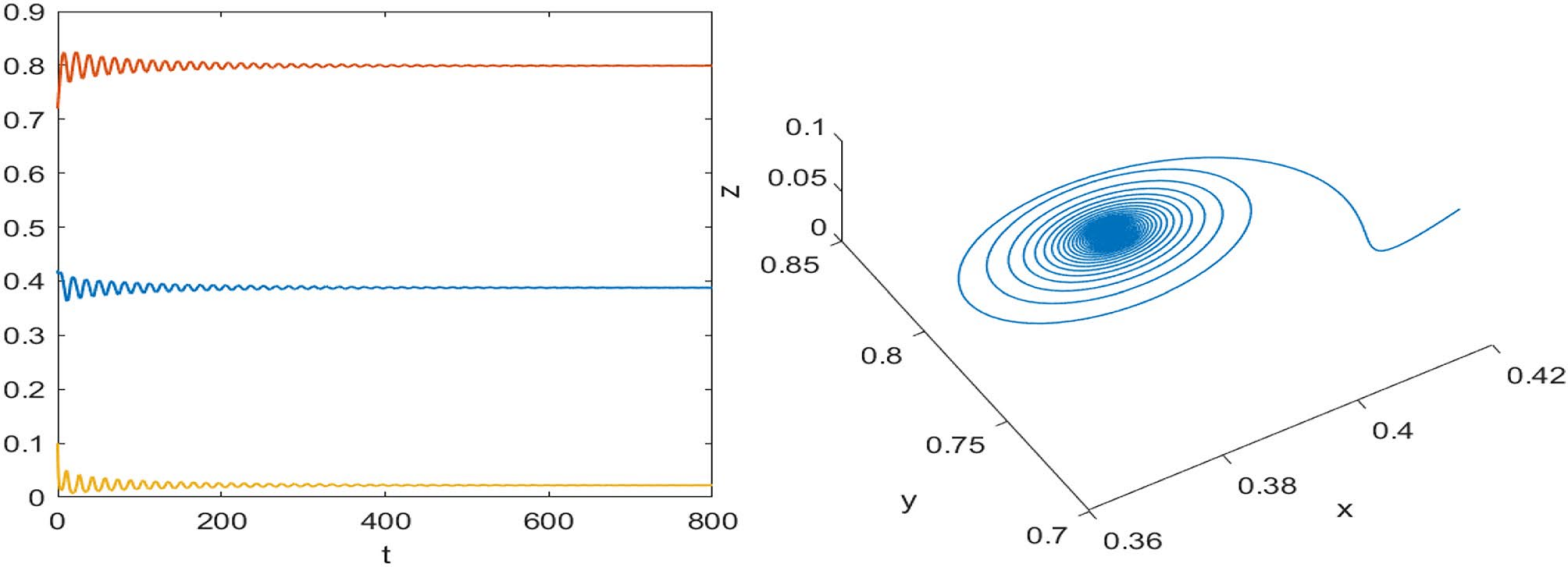
Time series and phase portrait of the system (3) for $\nu _1=0.069$ shows stable solutions in $\textbf {R}^3$ for the set of parameters (39).

**Fig. 11:**
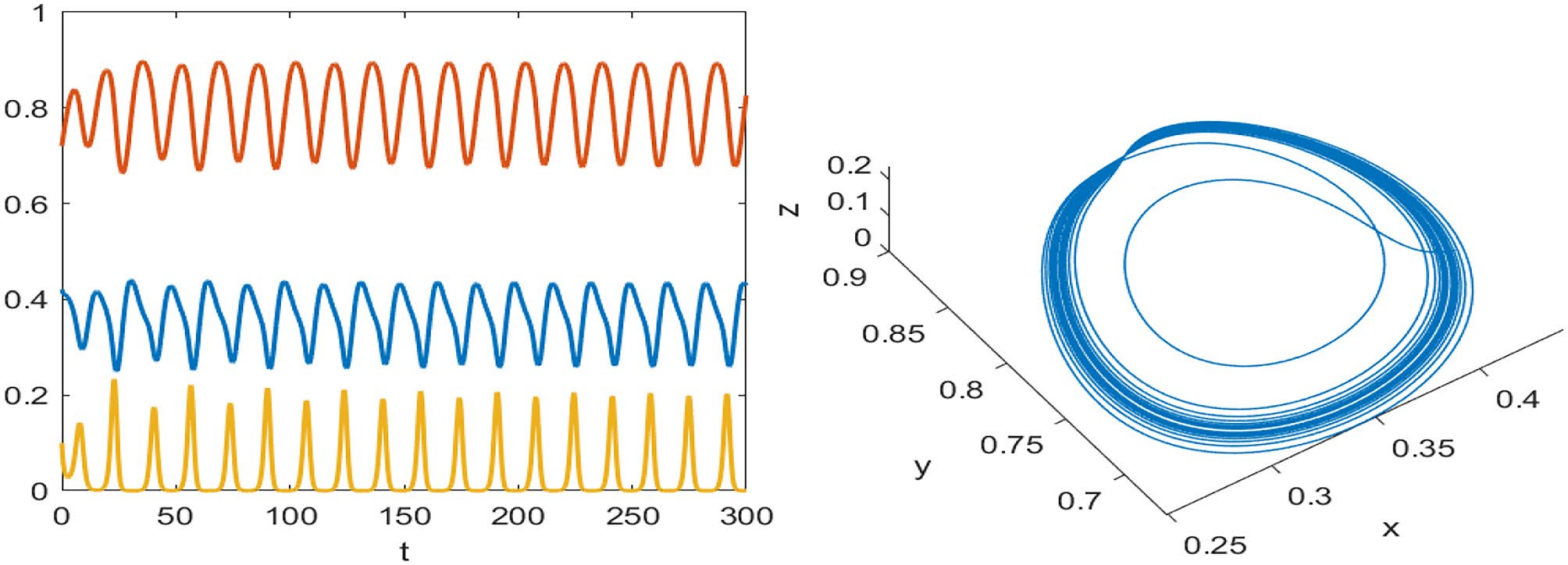
Time series and phase portrait of the system (3) around the interior point when $\nu _1=0.045$ gives periodic solutions for the set of parameters (39).

**Fig. 12:**
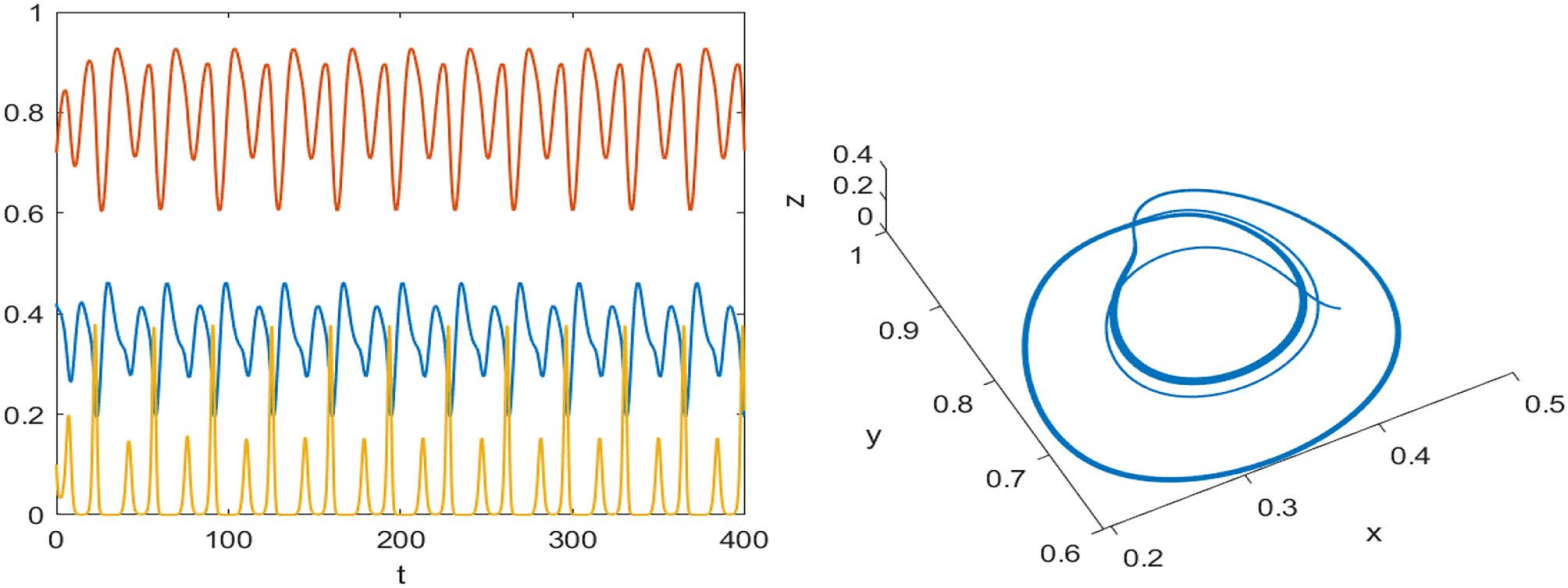
Time series and phase portrait of the system (3) around the interior point when $\nu _1=0.036$ gives period doubling solution of period-2 for the set of parameters (39).

**Fig. 13:**
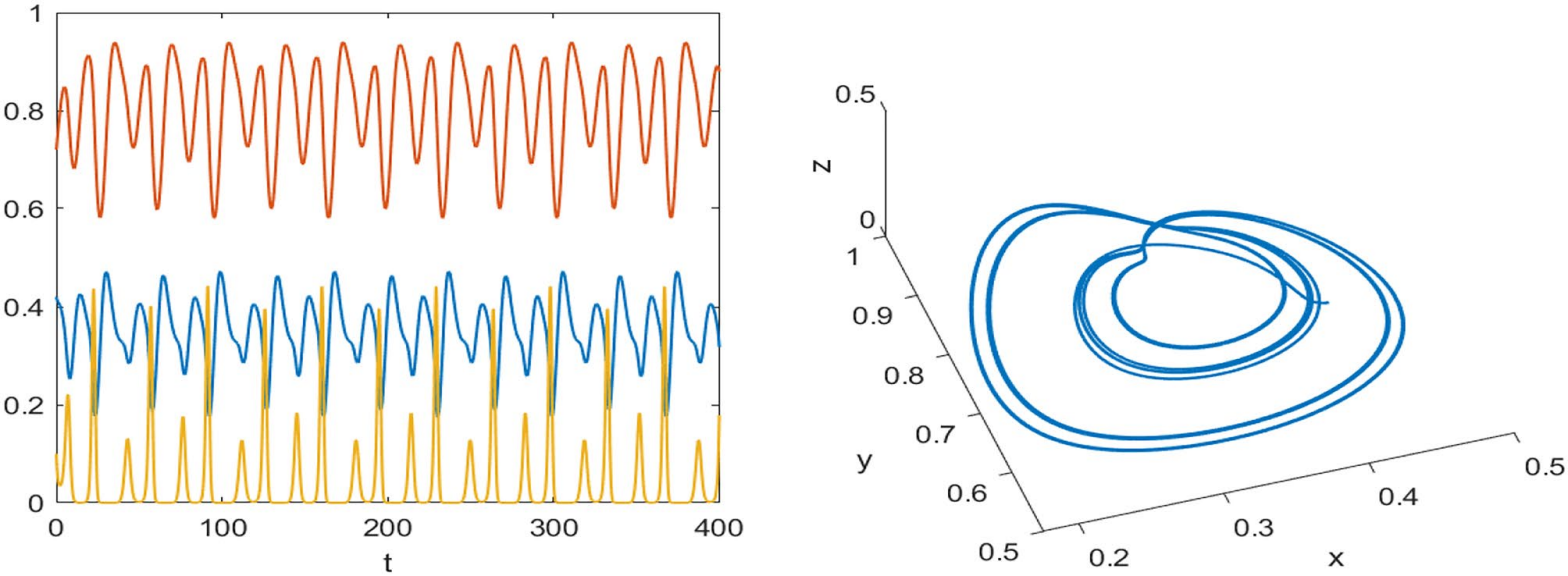
Time series and phase portrait of the system (3) around the interior point when $\nu _1=0.033$ gives period doubling solution of period-4 for the set of parameters (39).

**Fig. 14:**
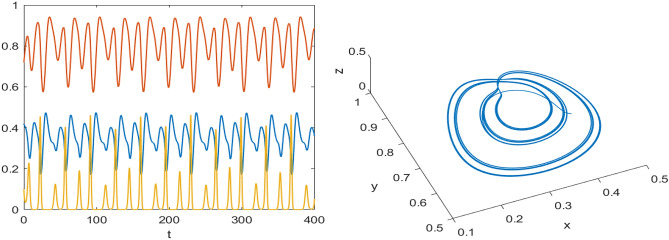
Time series and phase portrait of the system (3) around the interior point when $\nu _1=0.032$ gives period doubling solution of period-8 for the set of parameters (39).

**Fig. 15:**
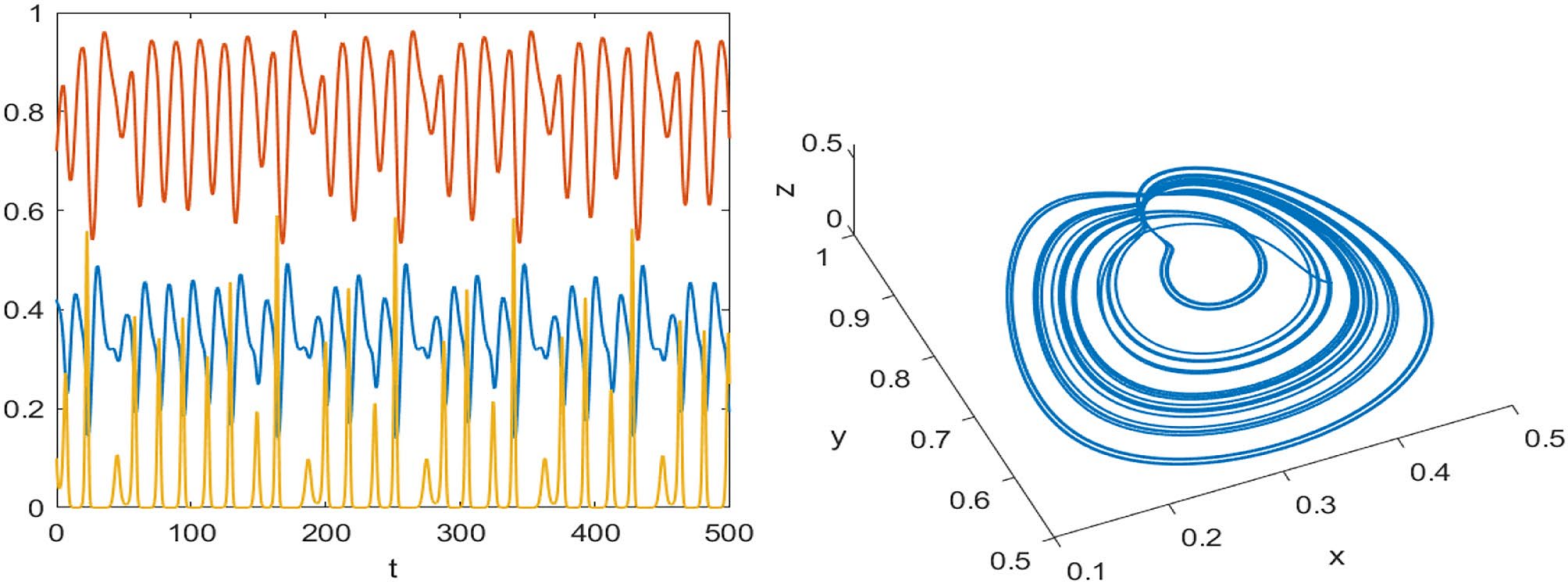
Time series and phase portrait of the system (3) around the interior point when $\nu _1=0.027$ shows chaotic solutions for the set of parameters (39).

**Fig. 16:**
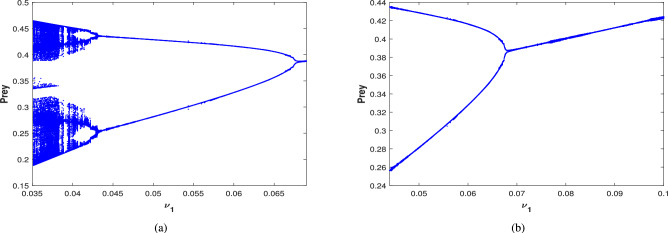
Bifurcation diagram of the system (3) with respect to $\nu _1$.

For the above given data set, it is evident that the trivial equilibrium point $P_0(0,0,0)$ is always saddle due the existence of positive eigenvalue. The boundary equilibrium point $P_1(1.11,0,0)$ is locally asymptotically stable as it met the condition (18). When $\nu _1=0.1$ and other parameters remain unchanged, the planar equilibrium point $P_2(0.4233,0,7951,0)$ has eigenvalues $(-0.2171+0.3700i,-0.2171-0.3700i,-0.0367)$, indicating that $P_2$ is locally asymptotically stable as it has negative real parts, as presented in Fig. 8. The interior equilibrium point $P^*(0.3881,0.8002,0.0218)$ is locally asymptotically stable for $\nu _1=0.069$, as displayed in Fig. 10. It is demonstrated that the interior point $P^*$ remains stable for $\nu _1\in (0.068,0.097)$ (see Figs. 9, 10). Our numerical analysis reveals the presence of a limit cycle (periodic solution) for $\nu _1\in (0.045,0.067)$, as illustrated in Fig. 11. A further decrease in $\nu _1$ results in period-doubling solutions: period-2 for $\nu _1\in (0.042,0.035)$, period-4 for $\nu _1\in (0.034,0.033)$, and period-8 for $\nu _1=0.032$, as shown in Figs. 12, 13, 14. Continued reduction in $\nu _1$ leads to a chaotic solution, with Fig. 15 depicting the chaotic behavior for the chosen parameters. From Fig. 16, the bifurcation diagram visually demonstrates the system’s dynamics changes w.r.t parameter $\nu _1$, highlighting the transitions from chaotic solutions to period-doubling, followed by periodic solutions, and ultimately achieving stability, as the harvesting rate of predator population increases.

**Fig. 17:**
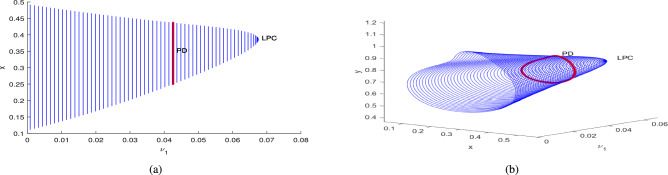
Two-dimensional (**a**) and three-dimensional (**b**) computation of limit cycle curve representation, started from the Hopf bifurcation point at $\nu _1=0.097250$.

The dynamical behavior of the system is analyzed using the MANTCONT software package by varying the parameters of the data set (39). This software helps in identifying bifurcations for the system (3) in the continuation of an interior point $P^*$. It reveals the presence of a Hopf point and branch point (BP), also known as transcritical bifurcation, as mentioned early in Figs. 2 and 3. In the continuation of the Hopf point with $\nu _1$ as a free parameter, the system (3) undergoes a limit point cycle (LPC), and the period-doubling bifurcation (PD) represents the period of limit cycle becomes 2-times and then $2^2$... and which are shown in the Fig. 17. The corresponding normal form coefficient for LPC is $-6.133386e^{-01}$ and for PD is $-1.833023e^{-05}$.

Moreover, the following Table 2 represent the equilibrium points and threshold values which are computed w.r.t the parameters $A_1, A_2$ and $A_3$, to determine the dynamical behavior changes in the Crowley–Martin functional response.

**Table 2:** A threshold values and equilibrium points of one parametric bifurcation w.r.t the parameters $A_1,A_2$ and $A_3$.

Parameters	Threshold values	The equilibrium points	$\sigma$ for Hopf-point
$A_1$	0.122553	$H_1=P^*(0.389404, 0.799242,0.023228)$	$-1.922793e^{+01}$
	1.335962	$H_2=P^*(0.677249, 0.792843, 0.033762)$	$-4.495316e^{+00}$
	1.514359	$BP=P_2(0.758722, 0.790183,0 )$	
$A_2$	0.137204	$H_1=P^*(0.389709,0.799240. 0.023445)$	$-1.906643e^{+01}$
	1.031391	$H_2=P^*(0.637496,0.794057,0.046612)$	$-4.253641e^{+00}$
	1.478445	$BP=P_2(0.758722,0.790183,0 )$	
$A_3$	0.416052	$H_1=P^*(0.389442 0.799242 0.023255)$	$-1.918821e^{+01}$
	1.822546	$H_2=P^*(0.639943 0.793984 0.045902)$	$-3.339711e^{+00}$
	2.177257	$BP=P_2(0.758722 0.790183,0 )$	

**Fig. 18:**
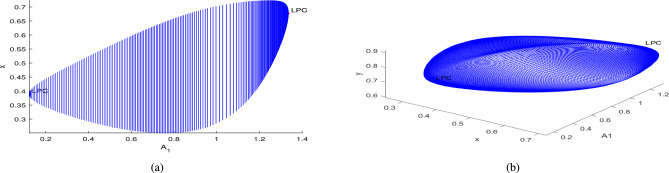
Two-dimensional (**a**) and three-dimensional (**b**) computation of limit cycle curve representation, started from the Hopf bifurcation point at $A_1=0.122553$.

**Fig. 19:**
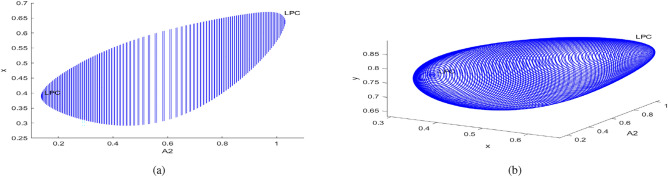
Two-dimensional (**a**) and three-dimensional (**b**) computation of limit cycle curve representation, started from the Hopf bifurcation point at $A_2=0.137204$.

**Fig. 20:**
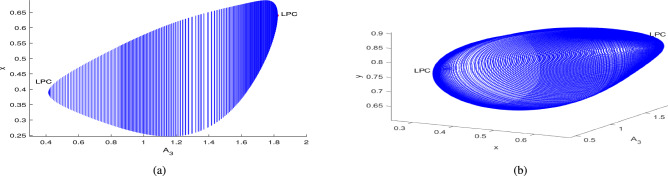
Two-dimensional (**a**) and three-dimensional (**b**) computation of limit cycle curve representation, started from the Hopf bifurcation point at $A_3=0.416052$.

In the continuation of first Hopf point with $A_1$ as free, the system (3) undergoes two limit point cycles (see in the Fig. 18). The corresponding normal form coefficient for first LPC is $4.664865e^{-01}$ and for second LPC is $-8.489392e^{-02}$. Similarly, in the continuation of first Hopf point with $A_2$ as free, the system (3) undergoes two limit point cycles (see in the Fig. 19). The corresponding normal form coefficient for first LPC is $2.199598e^{+00}$ and for second LPC is $-1.721856e^{-01}$. Similarly, in the continuation of first Hopf point with $A_3$ as free, the system (3) undergoes two limit point cycles (see in the Fig. 20). The corresponding normal form coefficient for first LPC is $-2.341853e^{+00}$ and for second LPC is $-1.193797e^{-01}$.

**Fig. 21:**
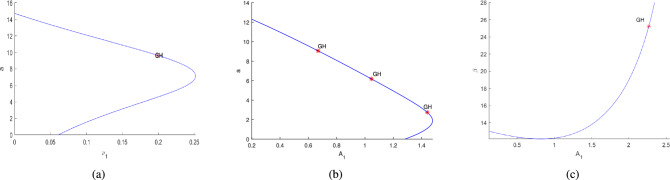
Detection of a generalized Hopf bifurcation points (GH) in different parametric planes are shown. The figure (**a**) shows the existence of a GH point along the continuation of Hopf point around at $\nu _1=0.067537$. The figures (**b**) and (**c**) indicate the existence of a GH point along the continuation of Hopf point around at $A_1=0.122553$.

**Fig. 22:**
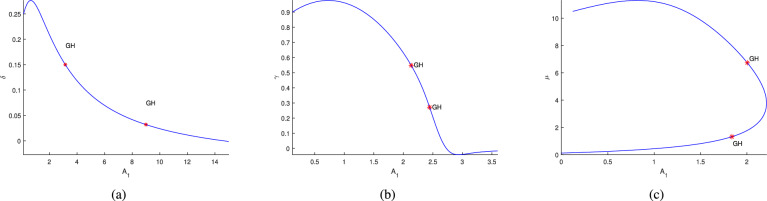
The figures (**a**)–(**c**) represent the existence of a GH point along the continuation of Hopf point around at $A_1=0.122553$.

**Fig. 23:**
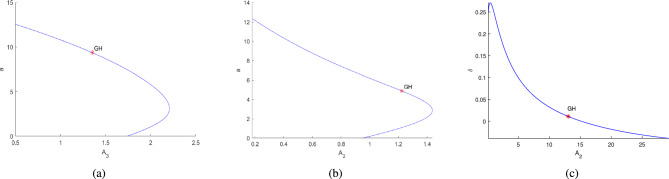
The figure (**a**) illustrates the existence of a GH point along the continuation of Hopf point around at $A_3=0.416052$. The figures (**b**) and (**c**) show the existence of a GH point along the continuation of Hopf point around at $A_2=0.137204$.

**Fig. 24:**
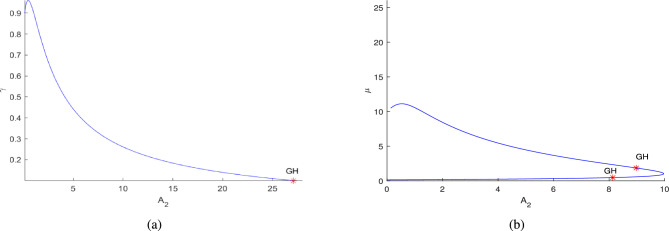
The figures (**a**) and (**b**) represent the existence of a GH point along the continuation of Hopf point around at $A_2=0.137204$.

Further, the numerical investigation of the system in co-dimension-2 parametric planes explored. Due to the system’s complexity, which makes its dynamics challenging to analyze analytically^16^. By continuing the Hopf point w.r.t the parameters $\nu _1$, $A_1$, $A_2$ and $A_3$, co-dimension-2 bifurcation points are detected in different parametric planes, as illustrated in Figs. 21a, 21b, 21c, 22a, 22b, 22c, 23a,23b, 23c, 24a, 24b, and are detailed in Table 3.

**Table 3:** Co-dimension-2 bifurcation points in various parametric planes obtained from the continuation of the Hopf point with respect to the parameters $\nu _1$, $A_1$, $A_2$, and $A_3$.

No. of generalized Hopf bifurcations (GH)	Bifurcation points	Figures
One	$(\nu _1,\alpha )$ = (0.198812,9.655609)	See in Fig. 21a
Three	$(A_1,\alpha )$ = (1.439968,2.747336)	
	$(A_1,\alpha )$ = (1.045442,6.178878)	See in Fig. 21b
	$(A_1,\alpha )$ = (0.668420,9.061650)	
One	$(A_1,\beta )$ = (2.268010,25.225792)	See in Fig. 21c
Two	$(A_1,\delta )$ = (3.149246,0.150113)	See in Fig. 22a
	$(A_1,\delta )$ = (8.99901,0.031967)	
Two	$(A_1,\gamma )$ = (2.133893,0.547867)	See in Fig. 22b
	$(A_1,\gamma )$ = (2.444613,0.271287)	
Two	$(A_1,\mu )$ = (2.004449,6.730729)	See in Fig. 22c
	$(A_1,\mu )$ = (1.837739,1.317796)	
One	$(A_3,\alpha )$ = (1.35323,9.367167)	See in Fig. 23a
One	$(A_2,\alpha )$ = (1.224037,4.881284)	See in Fig. 23b
One	$(A_2,\delta )$ = (13.046965,0.011099)	See in Fig. 23c
One	$(A_2,\gamma )$ = (28.60943,0.093699)	See in Fig. 24a
Two	$(A_2,\mu )$ = (8.976792,1.854898)	See in Fig. 24b
	$(A_2,\mu )$ = (8.134145,0.469444)	

**Fig. 25:**
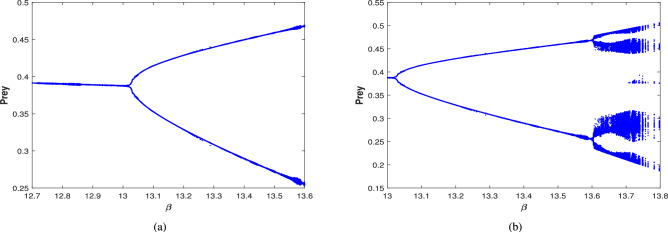
Bifurcation diagram of the system (3) in the existence of combined harvesting of predator and scavenger population i.e., $\nu _1\ne 0$,$\nu _2\ne 0$.

**Fig. 26:**
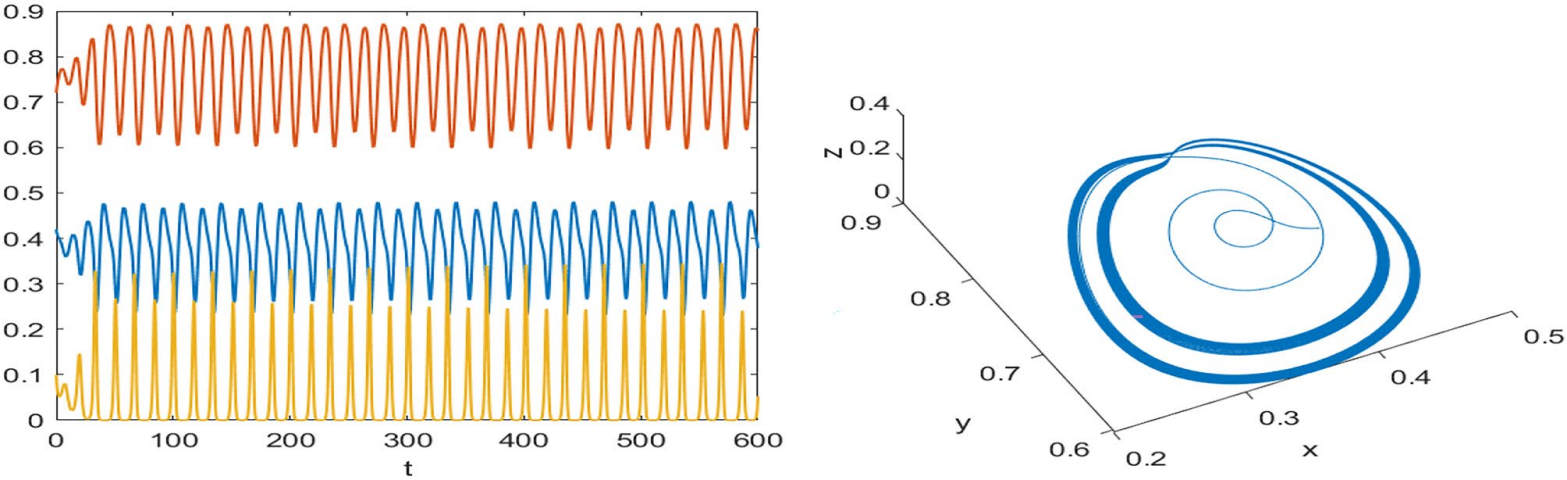
Time series and phase portrait of the system (3) when $\beta =13.65$ shows the period doubling solution with period-2.

Further, the effect of harvesting is studied for all possible cases for the given date set (39), with $\beta$ considered as a bifurcation parameter. The bifurcation diagram of system (3) is examined in the existence of combined harvesting of predator and scavenger population (i.e., $\nu _1\ne 0$,$\nu _2\ne 0$). As the benefit rates to the scavenger’s growth (from naturally died predator population) $\beta$ increases, the system loses its stability at a critical value of $\beta =13.03$, leading to a periodic solution for $\beta \in (13.03,13.59)$. This is followed by periodic doubling solution, with a period-2 solution emerging for $\beta \in (13.6,13.8)$, as illustrated in Fig. 25. The time series and phase portrait for the value of $\beta =13.65$ is drawn in Fig. 26, indicating there is a period doubling with period-2 cycle.

**Fig. 27:**
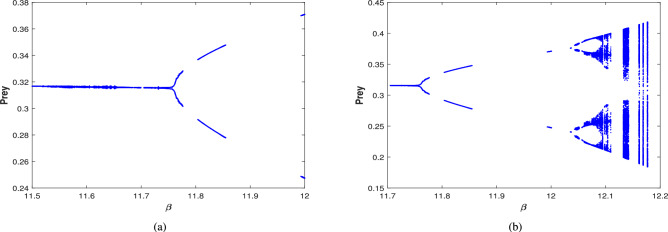
Bifurcation diagram of the system (3) in the nonexistence of harvesting of predator and scavenger population i.e.,$\nu _1=0$,$\nu _2=0$.

The bifurcation diagram in the nonexistence of harvesting of predator and scavenger population (i.e.,$\nu _1=0$,$\nu _2=0$) is also explored, which is presented in Fig. 27. This diagram indicates that as the value of $\beta$ increases, the system destabilizes at $\beta =11.77$ and transition into a periodic solution for $\beta \in (11.77,12.04)$. Then the system undergoes a period doubling with period-2 cycle at $\beta =12.1$, period-4 cycle at $\beta =12.15$, and period-8 cycle at $\beta =12.165$, eventually the system leading to chaotic dynamics for $\beta \ge 12.17$.

**Fig. 28:**
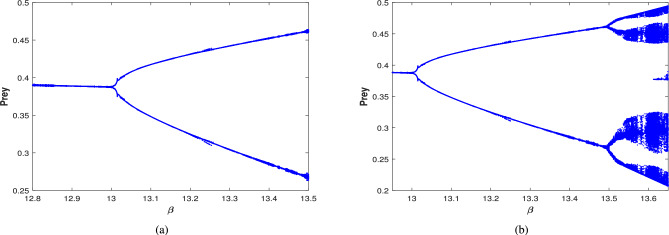
Bifurcation diagram of the system (3) in the presence of harvesting of predator population i.e., $\nu _1\ne 0$,$\nu _2=0$.

**Fig. 29:**
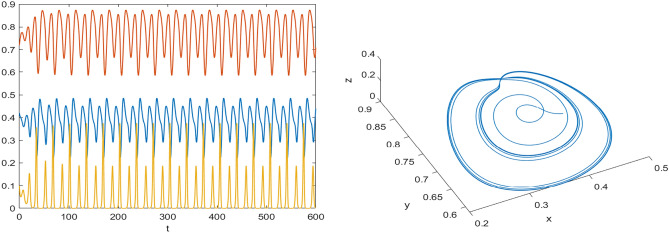
Time series and phase portrait of the system (3) when $\beta =13.6$ shows the period doubling solution with period-2.

**Fig. 30:**
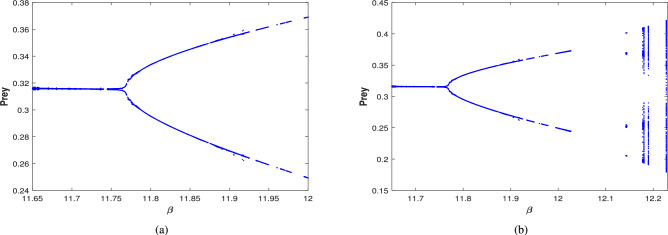
Bifurcation diagram of the system (3) in the presence of harvesting of scavenger population i.e., $\nu _1=0$,$\nu _2\ne 0$.

When only the predator population is harvested (i.e., $\nu _1\ne 0$,$\nu _2=0$), the bifurcation diagram of the system is depicted in Fig. 28. As $\beta$ increases, the system transition from stable to a periodic solution whenever $\beta \in (13.01,13.49)$. Further increase in $\beta$ resulting period doubling with period-2 cycle for $\beta \in (13.5,13.65)$. The time series and phase portrait for the system at $\beta =13.6$, which demonstrates a period-2 cycle, are shown in Fig. 29. Figure 30 illustrates the bifurcation diagram for the case where only the scavenger population is harvested (i.e., $\nu _1=0$,$\nu _2\ne 0$). As $\beta$ increases, the system loses stability at $\beta =11.77$, transitioning to a periodic solution for $\beta \in (11.77,12.09)$. Subsequently, period doubling occurs with period-2, period-4 and period-8 for $\beta \in (12.1,12.19),\beta \in (12.2,12.22)$ and $\beta =12.23$, respectively. Finally, chaotic dynamics emerges at $\beta =12.24$.

**Fig. 31:**
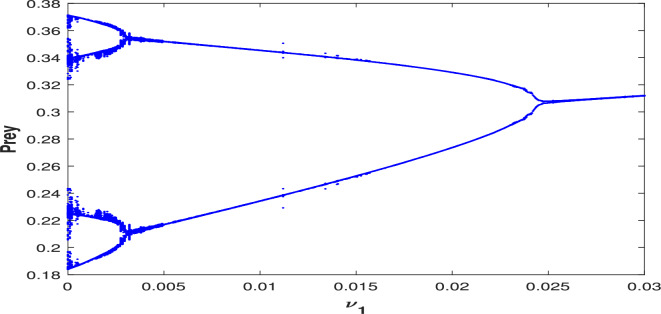
Bifurcation diagram of the system (3) with Holling-II functional response (i.e., when $A_3=0$ in the system (3)) w.r.t the bifurcation parameter $\nu _1$.

Additionally, the system’s dynamical behavior is analyzed by modifying the functional response from the Crowley–Martin functional response to a simpler Holling-II type. This can be achieve by setting the parameter $A_3=0$ in the system (3), using the data set (39). This alternation significantly affects the system’s dynamics, as seen in bifurcation diagram provided in Fig. 31. This diagram indicates that under these conditions, the system initially undergoes a period doubling with period-2 cycle for $\nu _1\in (0,0.0035)$. This is followed by a transitions to a periodic solution for $\nu _1\in (0.0036,0.024)$, after which the system stabilizes for $\nu _1\ge 0.025$, with no chaotic behavior observed.

## Optimal control problem

This section is completely devoted to develop a cost optimization problem related to harvesting. As, we are aware that harvesting needs man power and associated costs for a longer time period. In order to make a balance in implementing the harvesting efforts and to get more output, the optimal harvesting efforts is needed. In the model system (2), a constant harvesting efforts $E_1$ and $E_2$ of predator and scavenger respectively are considered. Therefore, for optimal control problem, we consider the constant harvesting effort rates $E_1$ and $E_2$ as time dependent dynamic control variables $E_1(T)$ and $E_2(T)$. The purpose is to find the optimal paths of $E_1$ and $E_2$ which maximize the total discounted net revenues. The corresponding cost functional for total revenue, for fixed time $T_f$, which need to be maximized is presented below

40 $$\begin{aligned} J= \int _0^{T_{f}} e^{-\delta _1 T}\left\{ \left( p_1-\nu _1 q_1 E_1(T) X_2^2\right) q_1 E_1(T) X_2^2+\left( p_2-\nu _2 q_2 E_2(T) X_3^2\right) q_2 E_2(T) X_3^2-C_1 E_1(T)-C_2 E_2(T)\right\} dT. \end{aligned}$$

subject to the model

41 $$\begin{aligned} \dfrac{dX_1}{dT}&= X_1(r-k X_1)-\dfrac{a X_1 X_2}{1+k_1 X_1+k_2 X_2+ k_3 X_1 X_2}-bX_1 X_3, \nonumber \\ \dfrac{dX_2}{dT}&= \dfrac{c X_1 X_2}{1+ k_1 X_1+k_2 X_2+ k_3 X_1 X_2}-d X_2- q_1 E_1(T) X_2 ^2, \nonumber \\ \dfrac{dX_3}{dT}&= l X_1 X_3 +m X_2X_3 -n X_3 -q_2 E_2(T) X_3 ^2. \end{aligned}$$

Here, the following integrand

$$\begin{aligned} L(X_1, X_2, X_3, E_1, E_2)&= e^{-\delta _1 T}\left( \left( p_1-\nu _1 q_1 E_1(T) X_2^2\right) q_1 E_1(T) X_2^2 \right. \\ &\quad \left. +\left( p_2-\nu _2 q_2 E_2(T) X_3^2\right) q_2 E_2(T) X_3^2-C_1 E_1(T)-C_2 E_2(T) \right) \end{aligned}$$

of the aforesaid cost functional represents the net discounted revenue. The parameter $\delta _1$ is annual discount rate and $\nu _1 , \nu _2$ are economic constants. Parameters $C_1$ and $C_2$ indicate the harvesting cost per unit effort,whereas $p_1$ and $p_2$ refer to the price of per unit biomass for the predator and scavenger population, respectively. The corresponding control set is defined as:

$$\begin{aligned} U=\{(E_1, E_2): 0 \le E_1\le E_{1max}, 0 \le E_2\le E_{2max}\}, \end{aligned}$$

where $E_1$ and $E_2$ are integrable functions.

### Existence of the optimal controls

Here, we show the existence of such optimal control functions which maximize the cost functional in finite time period. For this, we follow the results given in^32–34^. Since, the results given in these references are for minimization problem but our proposed problem is maximization one, therefore, first, we convert it into corresponding minimization problem as $maxJ[E_1, E_2]=-minJ[E_1, E_2]$. Thus, in the following, we discuss the existence of the optimal control functions for $J_1=-J$ with integrand $L=-L_1$.

#### Theorem 6

*There exists an optimal control pair* $E_1^*$ and $E_2^*$ *in* *U* *such that* $J_1[E_1^*, E_2^*]=min[J_1[E_1, E_2]]$ *corresponding to the control system* (40)–(41).

#### Proof

The conditions given in (Theorem 4.1 pp. 68 in^32^), must be satisfied for the existence of optimal controls which are discussed as:

(i) The set of solutions to the system (41) with control variables in *U* is non empty.
(ii) *U* is closed and convex and the state system can be written as linear function of control variables with coefficients depending on time and state variables.
(iii) Integrand $L(X_1, X_2, X_3, E_1, E_2)$ of the Eq. (41) is convex on *U* and $L(X_1, X_2, X_3, E_1, E_2)\ge g(E_1, E_2)$ where *g* is continuous and $\mid (E_1, E_2)\mid ^{-1}g(E_1, E_2)\rightarrow \pm \infty$ whenever $\mid (E_1, E_2)\mid \rightarrow \infty$, here $\mid . \mid$ represents the norm.

As discussed in previous sections, the solutions of the model system (41) are positive and bounded for each bounded control variables in *U*. Moreover, the right hand side functions of the system (41) follows the Lipschitz condition with respect to state variables. Thus, Picard–Lindelöf theorem^35^, ensures that solution set is not empty which leads to condition (i). Additionally, by definition, the control set *U* is closed and convex (condition (ii)). Furthermore, the right hand part of the model system (41) is linear in control variables $E_1$ and $E_2$ with coefficients depending on state variables only. Moreover, the integrand *L* is convex as the determinant of the corresponding Hessian matrix with respect to control variables $E_1$ and $E_2$.

Also, the integrand

42 $$\begin{aligned} L&=-e^{-\delta _1 T}\left( \left( p_1-\nu _1 q_1 E_1(T) X_2^2\right) q_1 E_1(T) X_2^2+\left( p_2-\nu _2 q_2 E_2(T) X_3^2\right) \right. \ \left. q_2 E_2(T) X_3^2 -C_1 E_1(T)-C_2 E_2(T) \right) \nonumber \\&\ge e^{-\delta _1 T}(P_1 E_1+P_2 E_1^2+P_3 E_2+P_4 E_2^2) \end{aligned}$$

where    $P_1=C_1-p_1q_1M_1^2$,   $P_2=\nu _1 q_1^2 M_1^2$,   $P_3=C_2-p_2q_2M_1^2$,    and    $P_4=\nu _2 q_2^2 M_1^2$,   with $M_1$ defined as the bound of $X_2$ and $X_3$.

Let $g(E_1, E_2)=e^{-\delta _1 T}(P_1 E_1+P_2 E_1^2+P_3 E_2+P_4 E_2^2)$ which is a polynomial in $E_1$ and $E_2$, and hence $\mid (E_1, E_2)\mid ^{-1}g(E_1, E_2)\rightarrow \pm \infty$ whenever $\mid (E_1, E_2)\mid \rightarrow \infty$ which ensures the condition (iii).

Hence, the existence of optimal control pair $E_1^*$ and $E_2^*$ is guaranteed using results in^32–34^ with $J_1[E_1^*, E_2^*]=min[J_1[E_1, E_2]]$. $\square$

### Characterization of optimal control paths

In this part, we focus to obtain the analytical forms of the applied controls. In order to characterize the analytical paths of controls, we first define the Hamiltonian as

43 $$\begin{aligned} H(X_1, X_2, X_3, E_1, E_2)=L(X_1, X_2, X_3, E_1, E_2)+\lambda _1 \frac{dX_1}{dT}+\lambda _2 \frac{dX_2}{dT}+\lambda _3 \frac{dX_3}{dT}, \end{aligned}$$

further, we apply the Pontryagin’s Maximum Principle^32,36^. Here, $\lambda =(\lambda _1,\lambda _2,\lambda _3)$ is referred to as as adjoint variable.

#### Theorem 7

*Let* $E_1^*$ *and* $E_2^*$ *denote the optimal control functions, with* $X_1^*,X_2^*$ and $X_3^*$ *representing the associated state variables for the control problem* (40)–(41). *Then there are adjoint variable* $\lambda =(\lambda _1,\lambda _2,\lambda _3)\in {\mathbb {R}}^3$ *that fulfill the subsequent canonical equations*:

44 $$\begin{aligned} \frac{d\lambda _1}{dT}&= \left( 2 k X_1+b X_3 +\frac{a X_2(1+k_2 X_2)}{(1+k_1 X_1+k_2 X_2+k_3 X_1 X_2)^2 }-r\right) \lambda _1 - \frac{C X_2(1+k_2 X_2)}{(1+k_1 X_1+k_2 X_2+k_3 X_1 X_2)^2 } \lambda _2-l X_3 \lambda _3,\nonumber \\ \frac{d\lambda _2}{dT}&= - e^{\delta _1 T}(2p_1q_1E_1X_2-4\nu _1q_1^2E_1^2X_2^3) + \frac{a X_1(1+k_1 X_1)}{(1+k_1 X_1+k_2 X_2+k_3 X_1 X_2)^2 }\lambda _1 \nonumber \\ &\quad -\left( -d-2 q_1 E_1 X_2\frac{C X_1(1+k_1 X_1)}{(1+k_1 X_1+k_2 X_2+k_3 X_1 X_2)^2}\right) \lambda _2-m_2 X_3 \lambda _3, \nonumber \\ \frac{d\lambda _3}{dT}&= - e^{\delta _1 T}(2p_2q_2E_2X_3-4\nu _2q_2^2E_2^2X_3^3) +bX_1 \lambda _1 -(lX_1+m_2X_2-n-2q_2E_2X_3)\lambda _3, \end{aligned}$$

*with transversality conditions* $\lambda _1(T_f)=0,\lambda _2(T_f)=0$ *and* $\lambda _3(T_f)=0$.

*The optimal controls* $E_1^*$ *and* $E_2^*$ *are provided as follows*,

45 $$\begin{aligned} {E_1}^*= min\left\{ max\left\{ 0, \dfrac{e^{\delta _1 T}(p_1 q_1 X_2^2-C_1)-\lambda _{2}q_1 X_2^2}{2e^{\delta _1 T}\nu _1 q_1^2 X_2^4}\right\} ,E_{1max}\right\} . \end{aligned}$$

*and*

46 $$\begin{aligned} {E_2}^*= min\left\{ max\left\{ 0, \dfrac{e^{\delta _1 T}(p_2 q_2 X_3^2-C_2)-\lambda _{3}q_2 X_3^2}{2e^{\delta _1 T}\nu _2 q_2^2 X_3^4}\right\} ,E_{2max}\right\} . \end{aligned}$$

#### Proof

Suppose $E_1^*$ and $E_2^*$ are the optimal control functions, and $X_1^*,X_2^*$ and $X_3^*$ are the corresponding optimal state variables for system (41) that minimize the cost functional (40). Then, by Pontryagin’s Maximum Principle, there are adjoint variables $\lambda _1,\lambda _2$ and $\lambda _3$ that meet the subsequent canonical equations

$$\begin{aligned} \frac{d\lambda _1}{dT}= -\frac{\partial H}{\partial X_1},\ \ \frac{d\lambda _2}{dT}= -\frac{\partial H}{\partial X_2}, \ \ \frac{d\lambda _3}{dT}= -\frac{\partial H}{\partial X_3} \end{aligned}$$

with transversality conditions $\lambda _1(T_f)=0, \lambda _2(T_f)=0$ and $\lambda _3(T_f)=0$. Here, Hamiltonian *H* is as given in (43).

Then, the (44) is justified.

From the optimality condition, we obtain

$$\begin{aligned} \frac{\partial H}{\partial E_i}=0, ~~~\text {at} ~~E_1=E_i^* \; \; \text {for} \; \; i=1,2. \end{aligned}$$

Thus,

$$\begin{aligned} E_1^*=\dfrac{e^{\delta _1 T}(p_1 q_1 X_2^2-C_1)-\lambda _{2}q_1 X_2^2}{2e^{\delta _1 T}\nu _1 q_1^2 X_2^4} \quad \text {and} \quad E_2^*=\dfrac{e^{\delta _1 T}(p_2 q_2 X_3^2-C_2)-\lambda _{3}q_2 X_3^2}{2e^{\delta _1 T}\nu _2 q_2^2 X_3^4}. \end{aligned}$$

Based on the characteristics of the control space *U* and preceding discussion, the optimal controls $E_1^*$ and $E_2^*$ are specified in (45) and (46). $\square$

**Fig. 32:**
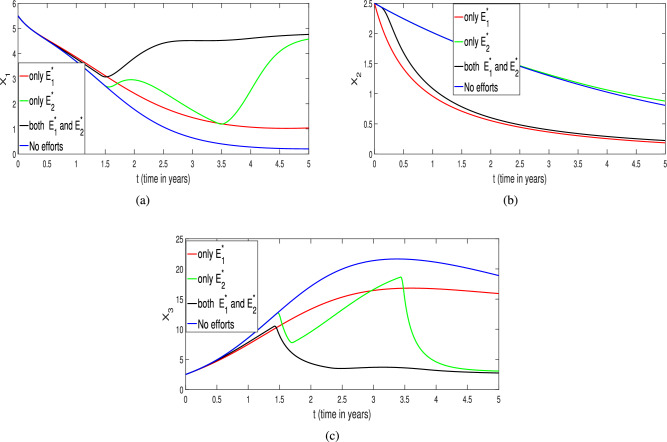
Profiles of the populations ($X_1, X_2$ and $X_3$ respectively) for various level of harvesting efforts. The red curve represents the implementation of effort $E_1^*$ only, the green curve shows the effect of applying effort $E_2^*$ only, the black curve corresponds to the simultaneous application of both efforts $E_1^*$ and $E_2^*$, and the blue curve illustrates the scenario with no control efforts.

**Fig. 33:**
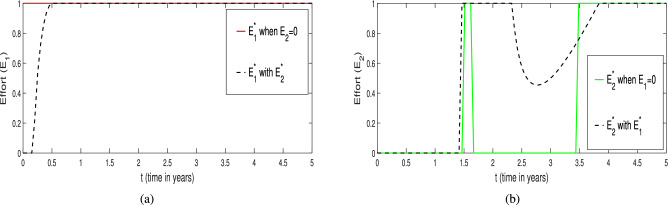
Profiles of optimal efforts $E_1^*$ and $E_2^*$ respectively. (**a**) The red solid line represents the control effort $E_1^*$ when $E_2 = 0$. The black dashed line corresponds to $E_1^*$ when both efforts $E_1^*$ and $E_2^*$ are applied together. (**b**) The green solid line shows the control effort $E_2^*$ when $E_1 = 0$. The black dashed line indicates $E_2^*$ when both efforts $E_1^*$ and $E_2^*$ are applied simultaneously .

**Fig. 34:**
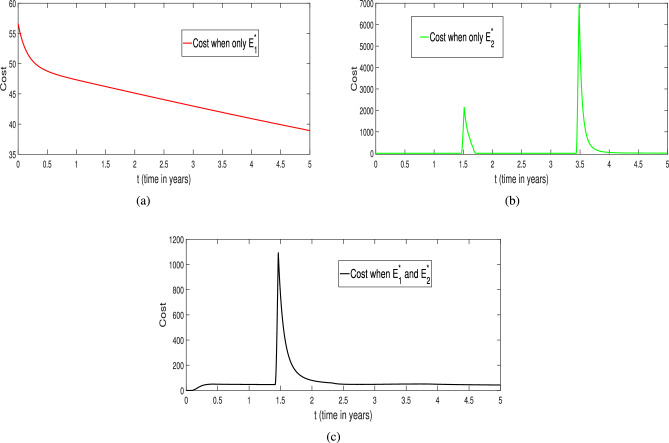
Profiles of the costs for various level of efforts. (**a**) The red curve indicates the cost profile when only the harvesting effort $E_1^*$ is applied. (**b**) The green curve shows the cost profile when only $E_2^*$ is implemented. (**c**) The black curve represents the cost profile when both $E_1^*$ and $E_2^*$ are applied simultaneously.

The following parametric values are taken for the optimal control problem: $r=5, k=0.9, \alpha =0.1, k_1=0.1,$ $k_2=0.12, k_3=0.48, b=0.25,$ $c=0.05, d=0.25, l=0.25, m_2=0.2,$ $n=0.35, q_1=0.5, q_2=0.5, \delta _1=0.05,$ $C_1=1, C_2=1, p_1=1,$ $p_2=1, \nu _1=1, \nu _2=1$ with $X_1(0)=5.5,$ $X_2(0)=2.5$ and $X_3(0)=2.5$ and the harvesting time period is $T_f=5$ years. To simulate optimality system (40)–(41), the standard forward-backward sweep method is employed. This approach begins with an initial for the optimal harvesting efforts and involves solving the optimal state system forward in time. Subsequently, the adjoint state system is solved backward in time, in accordance with the transversality conditions, using the values of optimal state variables and the initial guess for the optimal controls. The optimal control functions are then updated based on the optimal state variables and adjoint variables, and the process is repeated until a predefined convergence criterion is satisfied (for complete details see Ref.^37^). The numerical results obtained are illustrated in Figs. 32, 33 and 34.

The corresponding population profiles under different effort scenarios are given in Fig. 32. One can easily understand that how the different level the optimal efforts affect the dynamics of prey and predator species (blue and black color curves) with maintaining the optimal survival of any species. From Fig. 32a, the prey population is at very low level (blue curve) when no harvesting efforts are applied in predator and scavenger population whereas the prey population settles at an optimal level when both the efforts are applicable (black curve). At the same time, the coexistence or mutual survival of predator and scavenger population also take place with smaller capacity (black color curves in Fig. 32b, c) maintaining co-survival of all the species in ecosystem. Moreover, the execution of single harvesting effort either on predator or on scavenger population has its own impact of coexistence of all species (green and red color curves in Fig. 32). In between single harvesting efforts $E_1$ and $E_2$, the harvesting effort on scavenger population ($E_2$) plays a better role the harvesting effort $E_1$ (harvesting on predator population), in this case the prey population settles or survives with significant density. Thus, we found that the combined optimal harvesting efforts ($E_1$ and $E_2$) is highly effective in sustaining the prey population with high density along with low density of predator or on scavenger population in a biologically feasible environment. Therefore, the impact of comprehensive efforts is more suitable than any single effort policy (either $E_1$ or $E_2$).

The corresponding temporal profiles of the optimal harvesting efforts (controls) of $E_1^*$ and $E_2^*$ are depicted in Fig. 33. From Fig. 33a , it is clear that in the absence of the $E_2$ (harvesting of scavenger), the effort of harvesting on predator population ($E_1^*$, red color curve) has to be applied with full capacity for coexistence of species. Whereas, in the presence of the $E_2$, the effort of harvesting on predator population ($E_1^*$, black color curve) has to be applied with full capacity after around three months for coexistence of species optimally. A similar kind of pattern for the harvesting effort on scavenger ($E_2^*$) is found in the presence or absence of harvesting of harvesting on predator population ($E_1$) which is summarized as green and black color curves in Fig. 33b. Thus, finally, we conclude that the comprehensive effect of both the harvesting efforts ($E_1^*$ and $E_2^*$) plays a vital role in optimal coexistence of all the species with high density of prey population and also in single harvesting policies.

In addition, we have also captured the cost of each harvesting policy (single or combined one) and the corresponding results are given in Fig. 34. It is very clear from Fig. 34c that in the presence both harvesting efforts, the optimal cost sustains for entire period of consideration which gives the highly economical and feasible policy. Whereas, we found a bit different scenarios in the case of single harvesting efforts (either $E_1^*$ or $E_2^*$) as depicted in Fig. 34a, b. For example, if harvesting on predator ($E_1^*$) is applied, in the initial phase, we got huge profit but after some time it reduces gradually to maintain the optimal survival of all species together (red color curve in Fig. 34a). Whereas, in the case of $E_2^*$ only, we got a sustainable profit during entire period along with some economic hikes (green color curve in Fig. 34b). Overall, our study accentuates that the combined effect of both the harvesting efforts is highly effective and economical viable for optimal coexistence of all the species during the course of action.

**Fig. 35:**
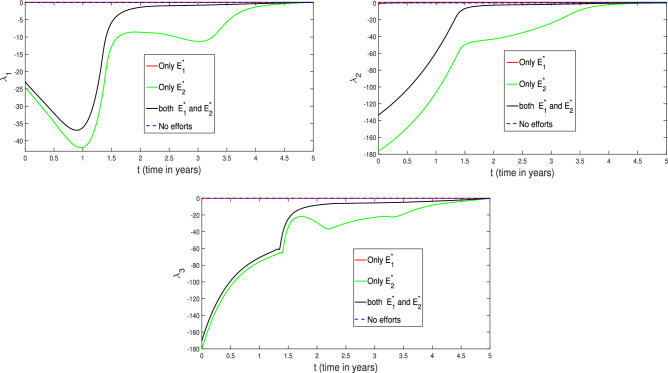
Profiles of the adjoint variables ($\lambda _1, \lambda _2, \lambda _3$) for various level of efforts. The red curve represents the implementation of effort $E_1^*$ only, the green curve shows the effect of applying effort $E_2^*$ only, the black curve corresponds to the simultaneous application of both efforts $E_1^*$ and $E_2^*$, and the blue curve illustrates the scenario with no control efforts.

Finally, we have also depicted the temporal distribution of the adjoint variables ($\lambda _1, \lambda _2, \lambda _3$) in the Fig. 35. Notice that the adjoint variables changes over time which have direct influence on efforts ($E_1$ and $E_2$) and hence these changes lead the variations in populations profiles.

## Conclusion

In this study, the dynamics of a three-species model involving prey, predator, and scavenger populations, incorporating the Crowley–Martin functional response and quadratic harvesting for predator and scavenger populations are investigated. The analysis ensures that the system remains positive, and bounded for positive initial conditions. The equilibrium points and their stability are determined, revealing complex behaviors such as limit cycles, period-doubling bifurcations, and chaotic dynamics. Further, the research reveals the presence of local bifurcations, including transcritical (BP) and Hopf bifurcations w.r.t the parameters $\nu _1$ and $\beta$, as well as global bifurcation (generalized Hopf bifurcations) under different parametric planes (See in Table 3). The system’s uniform persistence is also demonstrated under specific conditions, ensuring the long-term survival of the population. Numerical simulations corroborate the theoretical results, showing diverse dynamical behaviors through time series, phase portraits, and bifurcation diagrams. Notably, an increase in the harvesting rate of predator population ($\nu _1$), leads to the system stabilization.

The effects of harvesting on predator and scavenger populations are analyzed by considering different harvesting scenarios, leading to four distinct cases that influence system stability

(i) In the existence of combined harvesting of predator and scavenger population (i.e., $\nu _1\ne 0$,$\nu _2\ne 0$), an increase in the benefit rates to the scavenger’s growth ($\beta$) from naturally died predator destabilizes the system at a critical threshold , leading to periodic oscillations. This is followed by a period-doubling solution with period-2 solution. A similar outcome has been observed in the presence of predator population only (i.e., $\nu _1\ne 0$,$\nu _2=0$).
(ii) In the nonexistence of combined harvesting of predator and scavenger population (i.e., $\nu _1=0$,$\nu _2=0$), increasing $\beta$ causes the system instability, ultimately evolving into chaotic solution. A similar outcome has been observed when only the scavenger population is harvested (i. e., $\nu _1=0$,$\nu _2\ne 0$).

Further, an analysis under the Holling-II functional response, achieved by setting the parameter $A_3=0$ in the system, highlights significant variations in population dynamics, leading to a period doubling with period-2 solution.

To optimize harvesting strategies, an optimal harvesting problem is formulated to maximize the total revenue while minimizing harvesting efforts. Using Pontryagin’s maximum principle, analytical expressions for optimal harvesting efforts are derived, and conducted comparative numerical simulations. Results indicate that a combined harvesting approach yields the highest net revenue with minimal effort, making it the most efficient strategy within the given time frame. However, individual harvesting effort has its own importance during the harvesting period in maximizing the total revenue but with a lesser impact compared to combined efforts.

Overall, the results of this study have important ecological and economic significance, underscoring the complexity of prey–predator–scavenger interactions. The findings highlight the need to carefully manage ecosystems to avoid sudden population declines or unpredictable changes. It is important to adjust harvesting strategies based on the specific ecological conditions rather than using a fixed harvesting rates.

## Data Availability

All data analysed during this study are included in this published article.
